# Biophysics of high density nanometer regions extracted from super-resolution single particle trajectories: application to voltage-gated calcium channels and phospholipids

**DOI:** 10.1038/s41598-019-55124-8

**Published:** 2019-12-11

**Authors:** P. Parutto, J. Heck, M. Heine, D. Holcman

**Affiliations:** 1 Group of Data Modeling and Computational Biology, IBENS-PSL, Ecole Normale Supérieure, 75005 Paris, France; 2 Research Group Functional Neurobiology at the Institute of Developmental Biology and Neurobiology, Johannes Gutenberg University Mainz, Mainz, Germany; 3 DAMPT and Churchill College, University Of Cambridge, Cambridge, CB30DS United Kingdom

**Keywords:** Computational biophysics, Single-molecule biophysics, Biological physics, Scientific data

## Abstract

The cellular membrane is very heterogenous and enriched with high-density regions forming microdomains, as revealed by single particle tracking experiments. However the organization of these regions remain unexplained. We determine here the biophysical properties of these regions, when described as a basin of attraction. We develop two methods to recover the dynamics and local potential wells (field of force and boundary). The first method is based on the local density of points distribution of trajectories, which differs inside and outside the wells. The second method focuses on recovering the drift field that is convergent inside wells and uses the transient field to determine the boundary. Finally, we apply these two methods to the distribution of trajectories recorded from voltage gated calcium channels and phospholipid anchored GFP in the cell membrane of hippocampal neurons and obtain the size and energy of high-density regions with a nanometer precision.

## Introduction

Single Particle trajectories (SPTs) obtained from super-resolution techniques such as sptPALM or UPaint summarize the history of large amount of particles that can be cytoplasmic molecules, membrane receptors or channels in live cells. Over the past decade, statistical methods based on stochastic models have been developed to segment^1,2^, interpret and extract relevant biophysical parameters such as flows and arrival time statistics between various subregions^3–8^ from these large data sets. The most striking and universal characteristic of these trajectories is that they are not homogeneously distributed in cells, but rather are concentrated in sub-regions, a phenomenon that is not fully understood: what are these high-densities regions? What are the underlying physical forces that restrict and confine trajectories? For example, AMPA receptors that traffic on the surface of neuronal cells accumulate specifically at the post-synaptic density (PSD) of synapses, where they are needed for proper synaptic transmission^9,10^. Similarly, at the pre-synaptic terminal, voltage-gated calcium channels (CaV) can accumulate on membrane subregions, with a size of hundreds of nanometers^11^. Retaining these channels guarantee that calcium ions can remain near vesicles to trigger release.

A possible mechanism to retain trajectories is a field of force caused by the presence of an extended potential well. These structures have been detected in a size of hundreds of nanometers^3,11,12^. However, the physical origin of these wells remains unclear because the length of classical electrostatic interactions is ten time shorter^13^ than the observed wells sizes. These high-density regions are characterized by several features: (1) a converging field of force, whether or not it is the gradient of a potential energy, (2) an energy depth and (3) a boundary. Finding and estimating these geometrical characteristics from trajectories and their statistical distribution remain challenging especially at tens of nanometers below the diffraction limit of light.

Here, we present two methods to detect and reconstruct potential wells from high-density regions contained in SPTs. The first approach is based on estimating the density of points of a truncated Ornstein-Ulhenbeck process (which accounts for a motion driven by a converging force and diffusion). We recover the center of the well, the covariance matrix and the boundary. While the second approach is based on estimating the local drift vector field. We insist that the first approach will clearly reveal the peak of aggregation, while the strength of the second method is its ability to extract a field of force. This field confirms the underlying deterministic structure that maintains the random trajectories together. We will first validate both approaches on stochastic simulations and then apply them to characterize nanodomains appearing in voltage-gated calcium channels (CAV2.2) and lipid anchored GFP (GPI-GFP) trajectories obtained from sptPALM or UPaint experiments.

## Methods

### Coarse-grained description of stochastic trajectories

In the Smoluchowski’s limit of the Langevin equation^14,15^, the position (*t*) of a stochastic molecule at time t can be described by

1 $$\dot{X}=\frac{F({\boldsymbol{X}}(t),t)}{\gamma }+\sqrt{2D}\,\dot{W},$$

where *F*(***X***, *t*) is a field of force, *W* is a white noise and *γ* is the friction coefficient^14^ and *D* is the diffusion coefficient. The source of the noise is the thermal agitation of the ambient lipids and membrane molecules. However, due to the timescale of acquisition of trajectories, which is in general too low to follow the thermal fluctuations, rapid events are not resolved in data, and at this spatiotemporal scale, the motion can be coarse-grained as a stochastic process^3,16^

2 $$\dot{X}=a({\boldsymbol{X}})+\sqrt{2}B({\boldsymbol{X}})\dot{W},$$

where *a*(***X***) is the drift field and *B*(***X***) the diffusion matrix. The effective diffusion tensor is given by $D({\boldsymbol{X}})=\frac{1}{2}B({\boldsymbol{X}}){B}^{T}({\boldsymbol{X}})$(.^*T*^ denotes the transposition)^14,17^. The diffusion tensor accounts for impenetrable obstacles of various sizes. Note that the interpretation at the physical level of the stochastic Eq. (2) is from the Ito’s sense and not Stratanovich or any other sense, because a physical process has to be non-anticipating^17^ (the future cannot interfere with the past).

### Potential wells characteristics

The drift field *a*(***X***) in Eq. 2 may represent a field force acting on the diffusing particle, that could be due to a potential well^13^. When the diffusion tensor (***X***) is locally constant and the coarse-grained drift field *a*(***X***) is a gradient of a potential

3 $$a({\boldsymbol{X}})=-\,\nabla U({\boldsymbol{X}}),$$

then the density of particles is given locally by the Boltzmann distribution^18^

4 $$\rho ({\boldsymbol{X}})={N}_{0}{e}^{-U({\boldsymbol{X}})/D},$$

where *N*_0_ is a normalization constant. An infinite paraboloid potential well with an elliptic base has the analytical representation for ***X*** = (*x*, *y*)

5 $$U(x,y)=A({(\frac{x-{\mu }_{x}}{a})}^{2}+{(\frac{y-{\mu }_{y}}{b})}^{2}),$$

where the center is (*μ*_*x*_, *μ*_*y*_), *A* is the field amplitude *A* and *a*, *b* are the lengths of the large and small semi-axes of the ellipse. To account for a finite well, we restricted the influence of the well to the region

6 $${\Gamma }_{ {\mathcal E} }=\{(x,y)|U(x,y)\le  {\mathcal E} \}.$$

The truncated energy function *U* associated to such parabolic potential well is

7 $$U({\boldsymbol{X}})=\{\begin{array}{ll}A[{(\frac{x-{\mu }_{x}}{a})}^{2}+{(\frac{y-{\mu }_{x}}{b})}^{2}], & {\rm{if}}\,{\boldsymbol{X}}\in {\Gamma }_{ {\mathcal E} }\\  {\mathcal E}  & {\rm{otherwise}}\end{array},$$

from which the drift field is the gradient of the energy, is given by

8 $$\nabla U({\boldsymbol{X}})=-\,2A[\begin{array}{c}\frac{x-{\mu }_{x}}{{a}^{2}}\\ \frac{y-{\mu }_{y}}{{b}^{2}}\end{array}].$$

The goal of these section is to recover, from empirical single particle trajectories that consists of few successive points acquired with a sampling time Δ*t*, the center (*μ*_*x*_, *μ*_*y*_), the amplitude *A* and the size of each semi-axis *a*, *b* for the boundary ${\mathcal E}$.

### Simulations of stochastic trajectories

**Figure 1:**
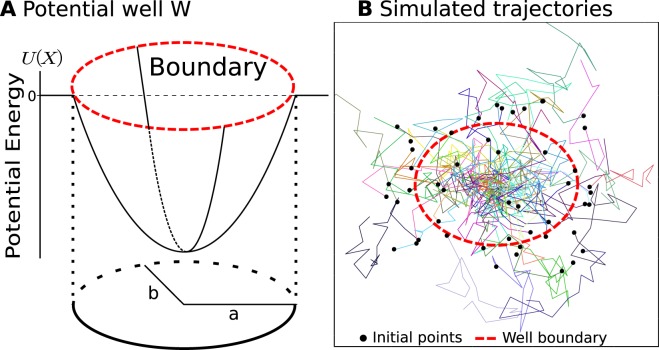
Numerical simulation scheme. (**A**) Model of a truncated potential well with two axes *a*, *b* and energy *U*(*X*) with a boundary. (**B**) Trajectories generated using Eq. (9) where the initial points (black dots) can either be located inside or outside the boundary of the well (dashed red). Parameters: *D* = 0.042 *μm*^2^/*s*, *λ*_*x*_ = 10,*λ*_*y*_ = 17.78.

To validate our methods, we first generated synthetic single particle trajectories from the stochastic process

9 $$\dot{{\boldsymbol{X}}}=-\,\nabla U({\boldsymbol{X}})+\sqrt{2D}\dot{W},$$

where the potential *U* is defined in Eq. (5) (as presented in Fig. 1A), *D* is the diffusion coefficient and *W* is a white noise. To reproduce observed trajectories, we keep a fixed lapse time Δ*t* between successive points and generated *N* trajectories (*X*_1_(0), …, *X*_*N*_(*K*Δ*t*)) containing *K* points (*K* = 20), using the classical Euler’s scheme (Fig. 1B).

We consider two types of numerical simulations depending whether the initial points *X*_*i*_(0) are uniformly distributed (1) inside the well or (2) inside a square box surrounding the well. This uniform distribution represents the random activation of fluorophores by a laser (Fig. 1B). To guarantee a constant number of points inside the wells across multiple simulations, we did not fix the number *N* of trajectories but instead generate new trajectories until a certain quantity of displacements has happened inside the well. This resetting procedure generates a distribution of points which depends on the initial uniform distribution. However, in the limit of large *N*, the distribution of points converges toward the steady-state, which is Gaussian inside the well and uniform outside, when trajectories are restricted to a large square domain.

### Empirical estimators

The drift of the stochastic model from Eq. 2 can be recovered from SPTs acquired at any infinitesimal time step Δ*t* by estimating the conditional moments of the trajectory displacements Δ*X* = *X*(*t* + Δ*t*) − *X*(*t*)^14,16,19–21^

10 $$a(x)=\mathop{\mathrm{lim}}\limits_{\Delta t\to 0}\frac{{\mathbb{E}}\,[\Delta {\boldsymbol{X}}(t)|{\boldsymbol{X}}(t)=x]}{\Delta t},$$

11 $$D(x)=\mathop{\mathrm{lim}}\limits_{\Delta t\to 0}\frac{{\mathbb{E}}[\Delta {\boldsymbol{X}}{(t)}^{T}\Delta {\boldsymbol{X}}(t)|{\boldsymbol{X}}(t)=x]}{2\Delta t}.$$

The notation ${\mathbb{E}}$ [·|*X*(*t*) = *x*] represents averaging over all trajectories that are passing at point *x* at time *t*. To estimate the local drift *a*(***X***) and diffusion coefficients *D*(***X***) at each point of the membrane and at a fixed time resolution Δ*t*, we use a similar procedure as the one for the estimation of the density in section 3 based on a square grid. The points of trajectories are first grouped within a lattice of squared bins *S*(*x*_*k*_,Δ*x*) centered at *x*_*k*_ and of width Δ*x* and the drift and local diffusion coefficient are estimated for each bin.

When there are *N* trajectories {*X*_*i*_(0),…, *X*_*i*_(*K*Δ*t*)}, with *i* = 1…*N* acquired with a sampling time Δ*t*, the discretization of Eq. 10 for the drift *a*(*x*_*k*_) = (*a*_*x*_(*x*_*k*_), *a*_*y*_(*x*_*k*_)) in a bin centered at position *x*_*k*_ is

12 $$\begin{array}{rcl}{a}_{x}({x}_{k}) & \approx  & \frac{1}{{N}_{k}}\mathop{\sum }\limits_{j=1}^{{N}_{t}}\,\mathop{\sum }\limits_{i=0,{x}_{j}(i\Delta t)\in S({x}_{k},r)}^{{N}_{s}-1}\,(\frac{{x}_{j}((i+1)\Delta t)-{x}_{j}(i\Delta t)}{\Delta t})\\ {a}_{y}({x}_{k}) & \approx  & \frac{1}{{N}_{k}}\mathop{\sum }\limits_{j=1}^{{N}_{t}}\,\mathop{\sum }\limits_{i=0,{x}_{j}(i\Delta t)\in S({x}_{k},r)}^{{N}_{s}-1}\,(\frac{{y}_{j}((i+1)\Delta t)-{y}_{j}(i\Delta t)}{\Delta t}),\end{array}$$

where *N*_*k*_ are the number of points of the trajectory falling in the square *S*(*x*_*k*_, *r*). Similarly, the components of the effective diffusion tensor *D*(*x*_*k*_) are approximated by the empirical sums

13 $$\begin{array}{rcl}{D}_{xx}({x}_{k}) & \approx  & \frac{1}{{N}_{k}}\mathop{\sum }\limits_{j=1}^{{N}_{t}}\,\mathop{\sum }\limits_{i=0,{x}_{j}(i\Delta t)\in S({x}_{k},r)}^{{N}_{s}-1}\,\frac{{({x}_{j}((i+1)\Delta t)-{x}_{j}(i\Delta t))}^{2}}{2\Delta t}\\ {D}_{yy}({x}_{k}) & \approx  & \frac{1}{{N}_{k}}\mathop{\sum }\limits_{j=1}^{{N}_{t}}\,\mathop{\sum }\limits_{i=0,{x}_{j}(i\Delta t)\in S({x}_{k},r)}^{{N}_{s}-1}\,\frac{{({y}_{j}((i+1)\Delta t)-{y}_{j}(i\Delta t))}^{2}}{2\Delta t}\\ {D}_{xy}({x}_{k}) & \approx  & \frac{1}{{N}_{k}}\mathop{\sum }\limits_{j=1}^{{N}_{t}}\,\mathop{\sum }\limits_{i=0,{x}_{j}(i\Delta t)\in S({x}_{k},r)}^{{N}_{s}-1}\,\frac{{x}_{j}((i+1)\Delta t)-{x}_{j}(i\Delta t))({y}_{j}((i+1)\Delta t)-{y}_{j}(i\Delta t))}{2\Delta t}.\end{array}$$

The bin centers and size Δ*x* are free parameters that should be optimized during the estimation procedure.

### Estimators for the elliptic boundary geometry

**Figure 2:**
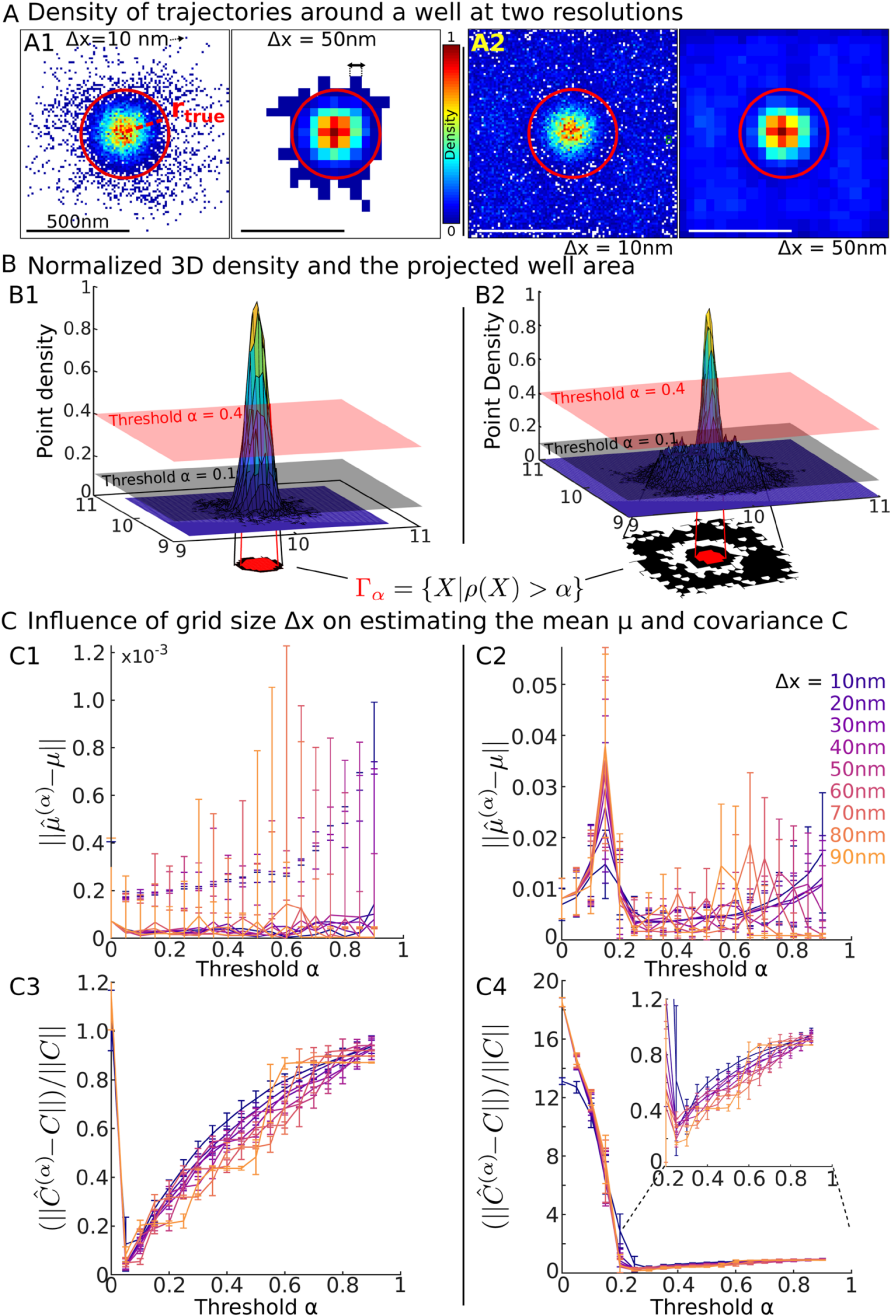
Recovering a truncated potential well from the density of points. (**A**) Density maps (in log(points)/*μm*^2^) for two different grid sizes Δ*x* = 10 (left) and 50 nm (right) when the initial points are located inside the well A1 or uniformly distributed in a square of size 1 *μm* A2. (**B**) Normalized three-dimensional empirical density function *ρ* obtained from A. We plotted the ensemble Γ_*α*_ = {*X*|*ρ*(*X*) > *α*} for *α* = 0.1 (black) and *α* = 0.4 (pink) and the projected area (red) in the well in the two cases (**B1**,**B2**) associated to (**A1**,**A2**) respectively. (**C**) Influence of the grid size Δ*x* and threshold *α* on the well characteristics estimations. (**C1**,**C3**) (resp. **C2**, **C4**) panels are obtained by computing with the initial distribution described in **A1** (resp. **A2**).

To identify parts of trajectories inside the well, we use the level line ensemble of the density distribution

14 $${\Gamma }_{\alpha }=\{{{\boldsymbol{X}}}_{i}\,{\rm{such}}\,{\rm{that}}\,{\rho }_{e}(x) > \alpha \},$$

where *ρ*_*e*_ is the empirical point density, estimated over the bins of the square grid constructed from the ensemble of trajectories (Fig. 2B). The ensemble Γ_*α*_ contains all trajectory points falling into a bin, with a density greater than the density threshold *α*.

To recover the center of the distribution, we consider all points ***X***_*i*_ = (*x*_*i*_, *y*_*i*_) located in Γ_*α*_ (Fig. 2C) and use the empirical estimators

15 $${\hat{\mu }}_{x}^{(\alpha )}=\frac{1}{{N}_{p}}\mathop{\sum }\limits_{\{k=1,{\boldsymbol{X}}{}_{k}\in {\Gamma }_{\alpha }\}}^{{N}_{p}}\,{x}_{k},\,{\hat{\mu }}_{y}^{(\alpha )}=\frac{1}{{N}_{p}}\mathop{\sum }\limits_{\{k=1,\,{{\boldsymbol{X}}}_{k}\in {\Gamma }_{\alpha }\}}^{{N}_{p}}\,{y}_{k},$$

where *N*_*p*_ is the number of points in the ensemble Γ_*α*_. To estimate the covariance two-by-two matrix *C*^(*α*)^, defined as

16 $$U({\boldsymbol{X}})={(-{\mu }^{(\alpha )})}^{T}{C}^{(\alpha )}(\,-\,{\mu }^{(\alpha )}),$$

we use the empirical estimators

17 $${\hat{C}}_{ij}^{(\alpha )}=\frac{1}{{N}_{p}-1}\mathop{\sum }\limits_{\{k=1,\,{{\boldsymbol{X}}}_{k}\in {\Gamma }_{\alpha }\}}^{{N}_{p}}\,{{\boldsymbol{X}}}_{i,k}{{\boldsymbol{X}}}_{j,k},$$

where ***X***_*i*,*k*_ is the *i*^*th*^ coordinates of ***X***_*k*_ (Fig. 2C).

### Improved drift estimation

We recall briefly here (see SI) that a correction term has to be added in order to recover an Ornstein-Uhlenbeck process of parameter *λ* and centered at *μ* (Eq. (9)): we derived in the SI that the drift term at position *x* and at resolution Δ*t*

18 $${\tilde{a}}_{\Delta t}(x)=-\,\frac{1-{e}^{-\lambda \Delta t}}{\Delta t}(x-\mu ).$$

Hence, the first order moment at resolution Δ*t* computed from the displacement *X*(*t* + Δ*t*) − *X*(*t*) from SPTs deviates from the expected drift. When *λ*Δ*t* is small, a first order Taylor expansion leads to the approximation

19 $${\tilde{a}}_{\Delta t}(x)=a(x)(1-\frac{1}{2}\lambda \Delta t)+o({\lambda }^{2}\Delta t)$$

and hence to recover the drift, we have to use the correction factor $1+\frac{1}{2}\lambda \Delta t$ on the estimated drift.

### Processing of CaV2.2 and GPI SPTs

For the experiments related to CaV2.2 data, we refer to^22^, while the experimental procedure of GPI-GFP data have been described for other molecules in^23^. We will first isolate trajectories in non-overlapping time windows of 20s and apply the following procedure to each window. We will construct a square grid with bins size Δ*x* around trajectories and collect the 5% highest density bins. For each of these selected bins, we will detect well as follows: we will first use 90% of the local point density (threshold *α* = 0.1) to detect the center of the well from Eq. (15), then we will apply the procedure described in subsection 3.1 (elliptic case), restraining the computation of the semi-axes ratio to a maximum distance from the center *r*_*cov*_ = 150 nm and using a threshold *T*_*ρ*_ = 35% on the density of points for determining ${\hat{r}}_{e,0}$. Once the center and semi-axes of the well are found, the diffusion coefficient will be determined using Eq. (13), estimated for all displacements with an initial points falling inside the well.

## Results

### Recovering a bounded potential well from the point density of trajectories

We first reconstruct the characteristics of the potential wells from the distribution of trajectories. This approach ignores the temporal causality between successive points and relies on a truncated paraboloid model. We will first recover the center and covariance matrix of the steady-state density distribution using a square grid (Fig. 2A). We recall that inside a well given by Eq. 5, this is a Boltzmann distribution:

20 $$\rho ({\boldsymbol{X}})={N}_{0}\,\exp \{-\frac{A({(\frac{x-{\mu }_{x}}{a})}^{2}+{(\frac{y-{\mu }_{y}}{b})}^{2})}{D}\},$$

where *N*_0_ is a normalization coefficient while the other parameters are defined in subsection 2.2. Based on this distribution, we estimated the center using Eq. (15) and the covariance matrix from Eq. (17).

The accuracies of these estimators are analyzed by plotting the errors between the true and the estimated centers $||{\hat{\mu }}^{\alpha }-\mu ||$ and between the covariance matrices $||{\hat{C}}^{\alpha }-C||$ (quadratic norm of the matrix) versus the parameter *α*, which represents the threshold of level line (Eq. 14, Fig. 2B,C) and various grid sizes (from Δ*x* = 10 to 90 *nm*). When *α* decreases from one to zero and the initial points are located inside the well, the iterative sequences of positions of the estimated centers converge to the true value and the fluctuations (SD computed over 100 realizations) decreases with *α* (Fig. 2C1). However, when the initial points of the simulated trajectories were also chosen outside the well, we found that there was an optimal threshold value *α* ≈ 0.3 for which the error in the estimated and true centers is minimum (Fig. 2C2). Below this value, points of the trajectories falling outside the well are also contained in the ensemble Γ_*α*_, thus contaminating the error of the estimation. When the initial points fall inside the well only (Fig. 2C3), the ensemble Γ_0_ contains external trajectories that perturb the estimation of the covariance matrix *C*^(*α*)^. However, as *α* increases, these external points disappear from Γ_*α*_ and the error becomes minimal at the value *α*_*opt*_ = 0.05. When *α* continues to increase, the estimators become less accurate. However, when the initial points are chosen also outside the well, the error starts by decreasing because trajectories that are not inside the well affects the estimation (Fig. 2C4). As *α* increases, the estimator converges toward an optimal value *α*0.25 (75% of the points are used), which minimizes the matrix error. When *α* continues to increase, the error increases slowly (Fig. 2C4-inset), similar to the case of Fig. 2C3.

To conclude, depending whether or not trajectories are falling inside the well or could also escape the high-density regions, the statistical estimators give different results: using as many points as possible increases the estimate of the center, but not necessarily of the covariance matrix.

#### Estimating the boundary of the well

**Figure 3:**
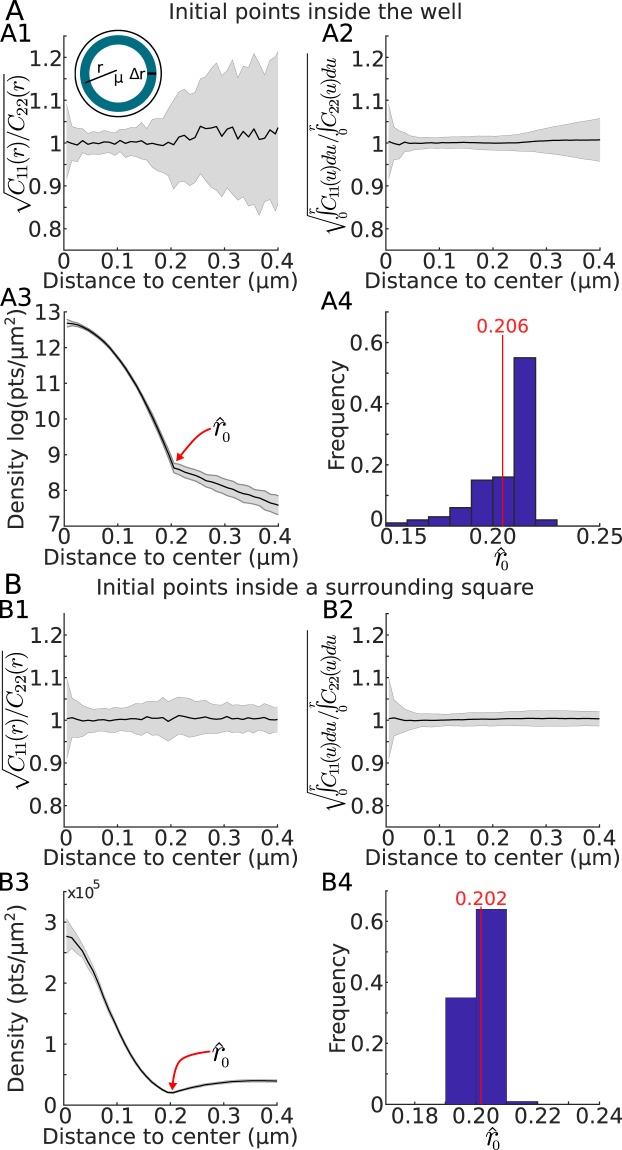
Estimating the potential well boundary. (**A**) Initial trajectory points are chosen inside the well while in (**B**) initial points are chosen inside a surrounding square. (**A1,B1**) Covariance ratio ${C}_{v}(r)=\sqrt{\frac{{C}_{11}(r)}{{C}_{22}(r)}}$ estimated in the annulus *r*, *r* + Δ*r*. (**A2,B2**) Cumulative from **A1,B1**. (**A3,B3**) Point density (in log for A3) estimated from the distance *r* to center showing a clear inflection point at the boundary of the well (criteria of selection). (**A4,B4**) Estimation of the radius ${\hat{r}}_{0}$ using the inflection point for **A4** (as presented in **A3**) or the minimum value of the density for **B4** (as presented in **B3**).

None of the estimators described above can be used to reconstruct the location of the well boundary. We now present a method to recover first a circular and then an elliptic boundary in two cases: when the initial points falls only inside the well and when they can also fall outside. The first step consists in discriminating between a circular and an elliptical boundary. To do so, we computed from the matrix (17), the covariance ratio

21 $$Cv(r)=\sqrt{\frac{{C}_{1,1}(r)}{{C}_{2,2}(r)}}$$

estimated over the trajectories located inside the annulus (*r*, *r* + Δ*r*) (Fig. 3A1). To compute *Cv*(*r*) (Fig. 3A1,A2,B1,B2), we recall that the diagonal form of covariance matrix can be found from Eqs. (20) and (16):

22 $$C=\frac{D}{A}[\begin{array}{ll}{a}^{2} & 0\\ 0 & {b}^{2}\end{array}].$$

Thus in that case, we expect that $Cv(r)=\sqrt{\frac{{C}_{1,1}(r)}{{C}_{2,2}(r)}}=\frac{a}{b}$, the ratio of the large to the small elliptic semi-axes lengths does not depend on any other parameters. In the case of a disk, *Cv*(*r*) = 1 as shown in the simulation cases (Fig. 3A1,A2,B1,B2).

Once the well boundary has been identified as circular, to estimate its radius *r*_0_, we plotted the density of points *ρ*(*r*) versus *r*, the radial distance with respect to the center $\hat{\mu }$ (see Method). Interestingly, this procedure reveals the location of the boundary between the Boltzmann (inside the well) and the uniform (surrounding it) density distributions of the trajectories (Fig. 3A3,A4). When the initial points falls inside the well, the density of points decays with the radius *r* and the boundary can be identified by plotting −log*ρ*(*r*) (Fig. 3A3). Indeed, for points inside the well, we have log*ρ*(*r*)~*C*_0_ − (*αx*^2^ + *βy*^2^), where *r*^2^ = *x*^2^ + *y*^2^, with *α* = 2*A*/*a*^2^, *β* = 2*A*/*b*^2^ and exp(*C*_0_) is the maximum value of the distribution. In practice, we find *r*_0_ as the first value for which the error ${\int }_{0}^{r}\,{({C}_{0}-{C}_{1}{s}^{2}+\log \rho (s))}^{2}ds$ starts to increase. The distribution of ${\hat{r}}_{0}$ for 100 simulations is shown Fig. 3A4. When the initial points are now also chosen outside the well, the trajectories are either attracted inside the well or leave, thus the distribution of points is minimal at the boundary (Fig. 3B3), which allows us to recover ${\hat{r}}_{0}$ as the minimum point of the density curve (Fig. 3B4).

**Figure 4:**
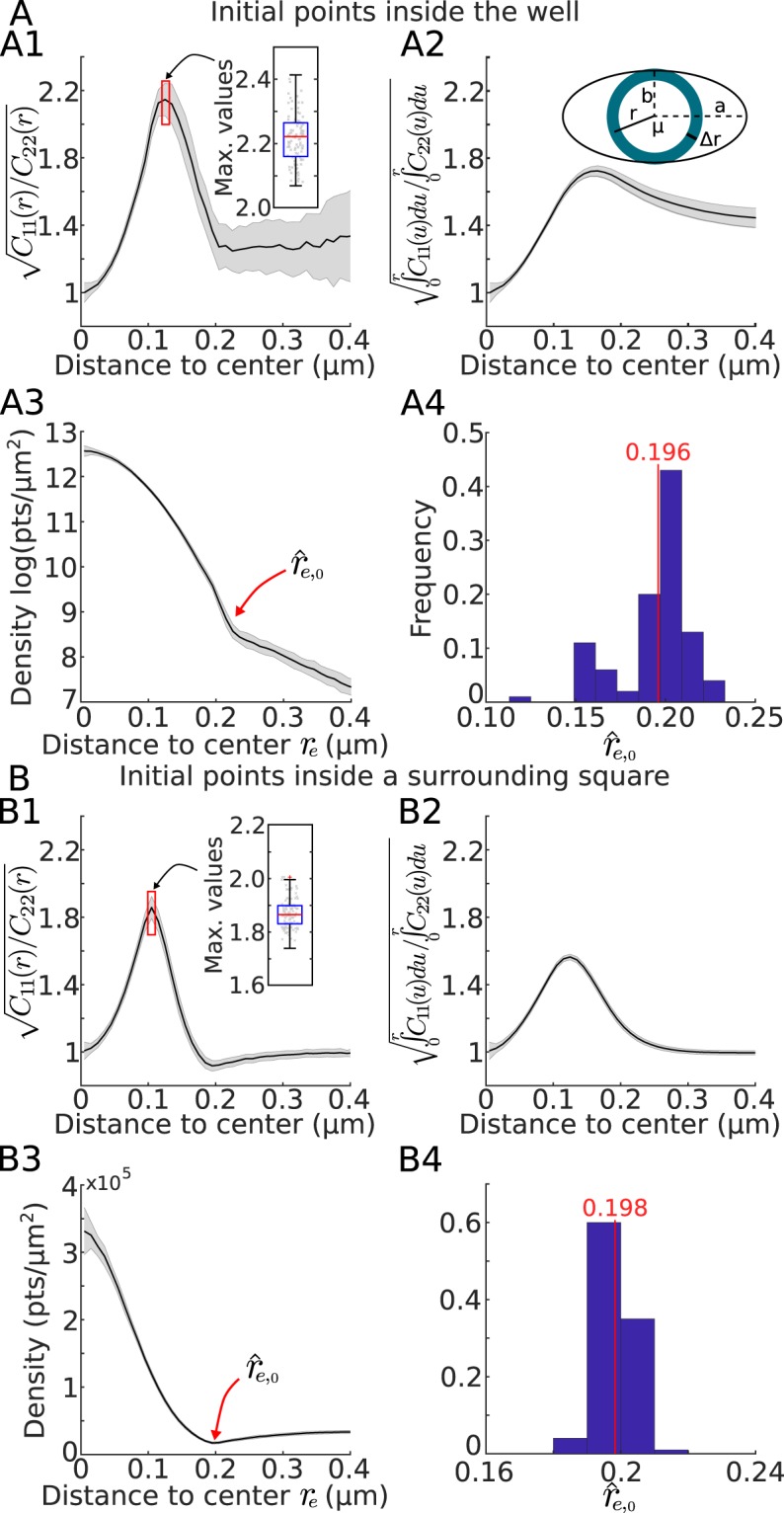
Estimating the potential well boundary for an elliptic well. (**A**) Initial trajectory points are chosen inside the well while in. (**B**) Initial points are chosen inside a surrounding square. (**A1,B1**) Covariance ratio ${C}_{v}(r)=\sqrt{\frac{{C}_{11}(r)}{{C}_{22}(r)}}$ estimated in the annulus *r*, *r* + Δ*r*. (**A2,B2**) Cumulative from A1,B1. (**A3,B3**) Point density (in log for **A3**) based on the modified distance ${r}_{e}(x,y)=\sqrt{{x}^{2}+{C}_{v}({r}^{\ast }){y}^{2}}$ to the center showing a clear inflection point at the boundary of the well. (**A4,B4**) Estimation of the radius ${\hat{r}}_{e,0}$ using the inflection point for **A4** (as presented in **A3**) or the minimum value of the density for **B4** (as presented in **B3**).

In the case of an elliptic well, we modified the previous method as follows: first, the ratio of the semi-axes lengths *a*/*b* is recovered as the maximal value of *Cv*(*r*) (Fig. 4A1,A2,B1,B2, for a ratio *a*/*b* = 2). Second, using this ratio, we introduced the elliptic distance ${r}_{e}(x,y)=\sqrt{{x}^{2}+{C}_{v}({r}^{\ast }){y}^{2}}$, for a point *P* = (*x*, *y*) from which we generated the point density distribution (Fig. 4A3,B3) and used on this curve the procedure described for the disk case to recover the large semi-axis $\hat{a}={\hat{r}}_{e,0}$ (Fig. 4A4,B4). The small-semi axis is then given by $\hat{b}=\frac{\hat{a}}{\sqrt{{C}_{v}({r}^{\ast })}}$.

To conclude, the present method based on the density of points allows to reconstruct the geometrical parameters of a bounded parabolic potential well: center, boundary, small and large semi-axes. In SI Figs. S1 and S2, we compare this density method with the MLE, which is classically used to recover the center and covariance, but not the boundary.

### Estimating the characteristics of the well using the velocity distribution

In this section, we describe a second approach to reconstruct the potential well associated to a nanodomain, using the statistics of displacements *X*(*t* + Δ*t*) − *X*(*t*). They allow to recover the drift of the vector field and reconstruct the center *μ* and the two axes *a*, *b* of the well boundary. This method is based on the least square quadratic error (LSQE),

23 $$Er{r}_{{\boldsymbol{b}}}({\mu }_{x},{\mu }_{y},{\lambda }_{x},{\lambda }_{y})=\mathop{\sum }\limits_{i=1}^{N}\,{\Vert -\nabla U({X}_{i})-({X}_{i})\Vert }^{2}$$

between the empirical drift and the parabolic well *U*, defined in Eq. 8, with ${\lambda }_{x}=-\,\frac{2A}{{a}^{2}},\,{\lambda }_{y}=-\,\frac{2A}{{b}^{2}}$.

#### Estimating the center and the field coefficients of the potential well

**Figure 5:**
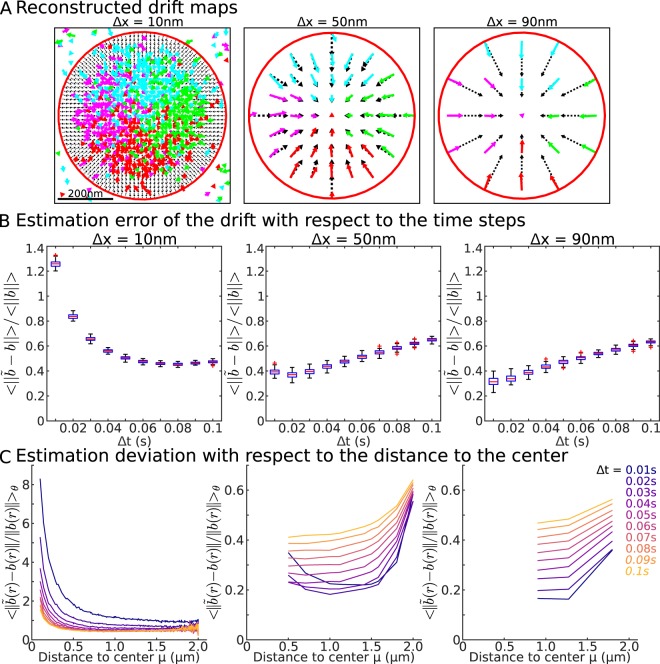
Vector field characteristics. (**A**) Recovering the local drift field inside a circular well for different grid sizes (10 nm, 50 nm, 90 nm) using numerical simulations with Δ*t* = 20 ms, with the constraints that at least 10 points falls inside a bin. (**B**) Error between the true and observed fields averaged over all the square bins inside the well vs the time step Δ*t*. (**C**) Error between the true and observed fields averaged over the radial angle vs the distance r to the center of the well center for various timestep (see color code).

The center *μ* and the coefficients *λ* of the potential well can be obtained explicitly from Eqs. 24 and 20 (SI). We compare in Fig. 5A, the reconstructed and the true drift value based on Eq. (12) for various grid sizes. At this stage, we considered the boundary to be known and estimated the drift only for bins that are falling inside the well. The error of the norm $\frac{\langle ||\bar{b}-b||\rangle }{\langle ||b||\rangle }$ is plotted in Fig. 5B for multiple time steps Δ*t* and for three grid sizes Δ*x* = 10, 50 and 90 nm. Having both a small grid size and time step Δ*t* produces a large error that quickly decreases with increasing the time step Δ*t*. Interestingly, for a large grid size, we found a slow increase of the error when increasing Δ*t*. To better understand which parts of the field contributed the most to the error, we plotted *Err* versus the distance to the center (Fig. 5C). This result shows that for small size Δ*x* = 10 nm, a major contribution came from the center, while for large step Δ*x* = 50, 90 nm, an error came also from the boundary. We refer to Fig. S3 for recovering a drift at a different time resolution Δ*t* and also with some restrictions on the trajectories for which the end point remained inside the well (Fig. S4).

**Figure 6:**
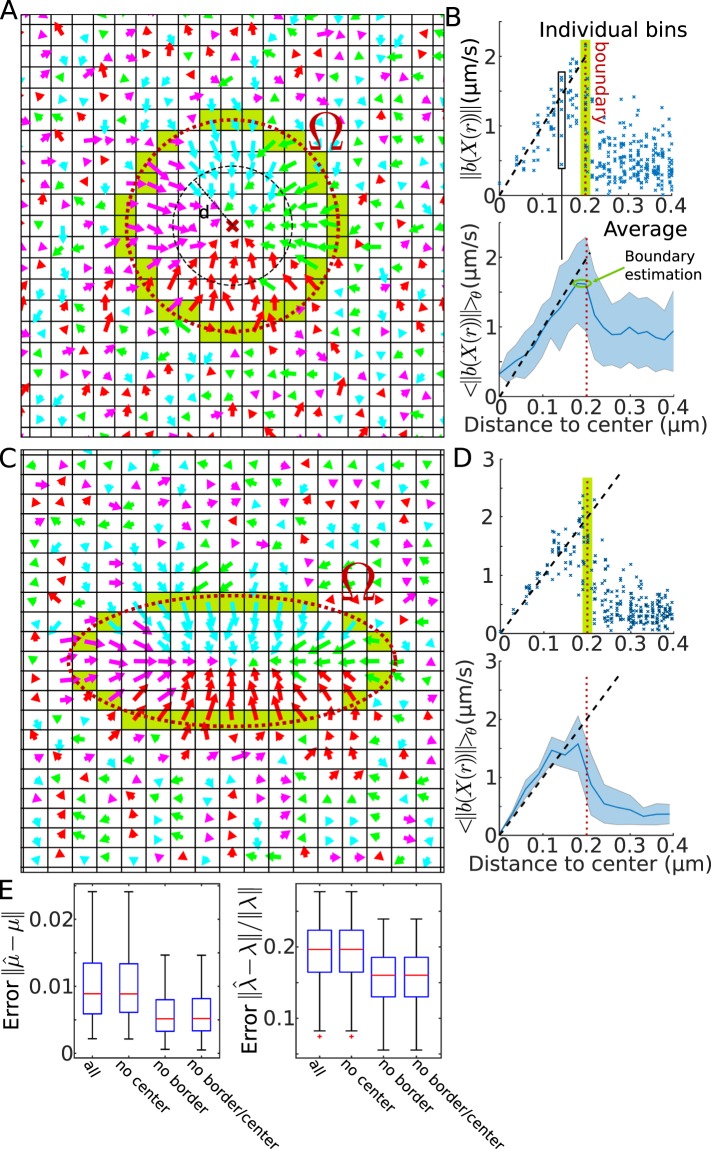
Recovery of the well depth from trajectories. (**A**) Vector field recovered from trajectories including the field generated by the Brownian dynamics outside the well. At the boundary of the well, there are mixed displacements (OU and Brownian), marked by the green band. (**B**) Upper: Drift amplitudes in each grid square versus the distance *r* to the center of the well. The expected amplitude is marked by a dashed line and the boundary with a narrow green band. Lower: Average and SD of the upper panel. (**C**,**D**) Same as in A-B for the case of an ellipse where *a*/*b* = 2. (**E**) Error of the center $||\mu ||=\sqrt{{\mu }_{x}^{2}+{\mu }_{y}^{2}}\,\,$and the eigenvalue $||\lambda ||=\sqrt{{\lambda }_{x}^{2}+{\lambda }_{y}^{2}}$ of the reconstructed ellipse in 4 cases: (1) bins falling inside the boundary are considered, (2) the center bin has been omitted (3) boundary bins are omitted and (4) when the center bin plus the ones intersecting the boundary (green bin in **C**) are not considered.

Finally, to estimate the boundary of the well from the drift distribution (Fig. 6A), we plotted the drift amplitude versus the distance to the well center (Fig. 6B, blue crosses representing the drift amplitude in individual bins). From the distribution and the average (Fig. 6B lower panel), we could recover the location of the boundary at the local maximum. Indeed, after the boundary is passed, the contribution of the deterministic field disappears and only fluctuations due to the Brownian motion remains in the statistics. We apply the same procedure for the case of an ellipse (Fig. 6C,D) and recover the boundary after we used the covariance ratio *Cv* (Eq. 21) to plot the drift amplitude versus the elliptic distance to the boundary.

To evaluate the influence of the bins located at the center or the ones near the boundary, we estimated the center *μ*, and eigenvalues *λ*_*x*_ and *λ*_*y*_ in four cases: for all bins falling inside the well, all bins except the ones at the center, all bins except the ones intersecting the boundary and finally removing the center and the boundary bins (Fig. 6E). We found that the latter case produces the best estimation.

### Interpretation high-density regions for CaV and GPI-GFP as potential wells

In this section, we will apply the methodology developed in the present article to characterize high-density regions found in SPTs of voltage-gated calcium channels and phospholipids. We recently reported that these regions could be associated with potential wells, as revealed from the voltage-gated calcium channels CaV2.1 isoform^11^. We focus here on the isoform CaV2.2 (N-type channel) by using the density of points, the least-square estimation (SI Section 2) and the maximum-likelihood method (SI section 1). For the analysis, we use only wells that contain at least 50 points with a minimum of 5 different trajectories.

**Figure 7:**
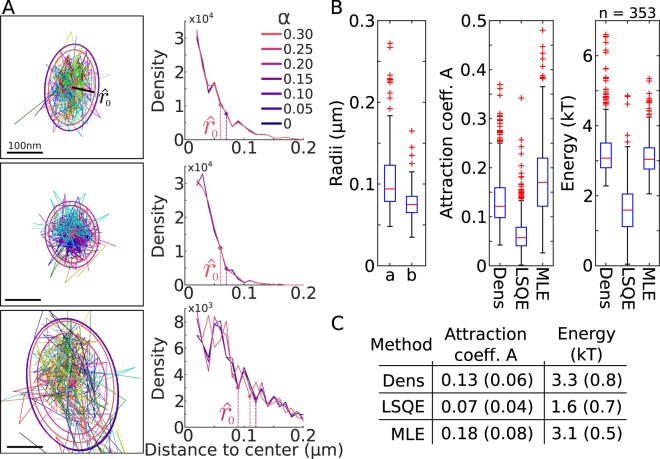
Reconstruction of wells associated to CaV2.2. (**A**) 3 examples of potential wells (left) obtained from the density analysis on SPTs. The boundary of the well are estimated from various level of density *α* (right). The estimated radius ${\hat{r}}_{0}$ is obtained using a threshold *T* = 4% on the density. (**B**) Box plots for the statistics computed over 353 detected wells for the two semi-axes *a* and *b* of the ellipse, the coefficient *A* and the energy (in kT). Results are obtained for the Density, LSQ and MLE methods. (**C**) Summary of mean and SD for the coefficient *A* and the energy.

We find that all three approaches produce reasonable values of the coefficient *A* and the energy (we restricted to wells with energies <7 kT). The values of the parameters are summarized in Table 1. We report in Fig. 7A–C that the high-density regions can be characterized as potential wells with the following characteristics: the two main axes have average lengths (±SD) of *a* = 104 ± 36 nm, *b* = 77 ± 20 nm associated with a mean energy of 3.3 kT estimated for the density method. These results differ from the CaV2.1 isoform^11^. Note that the distribution of energy varied with the statistical method (Fig. 7C), as we reported *E* = 3.1 ± 0.5 kT for the MLE and *E* = 1.6 ± 0.7 kT for the LSQ. To conclude, this statistical analysis suggests that to trap calcium channels, specific long-range molecular mechanisms should be present in the active zone of the pre-synaptic terminal, probably associated to vesicular release molecules such as synaptotagmin. These sites retain channels for a long time, enough to trigger vesicular release.

**Table 1:** Parameters used for CaV and GPI analysis.

Parameter	GPI dataset	CaV dataset
Δ*t* (exp)	20 ms	33 ms
Δ*x*	40 nm	30 nm
*r*_*min*_	30 nm	20 nm
*r*_*max*_	300 nm	400 nm
Δ*r*	20 (m	10 nm

**Figure 8:**
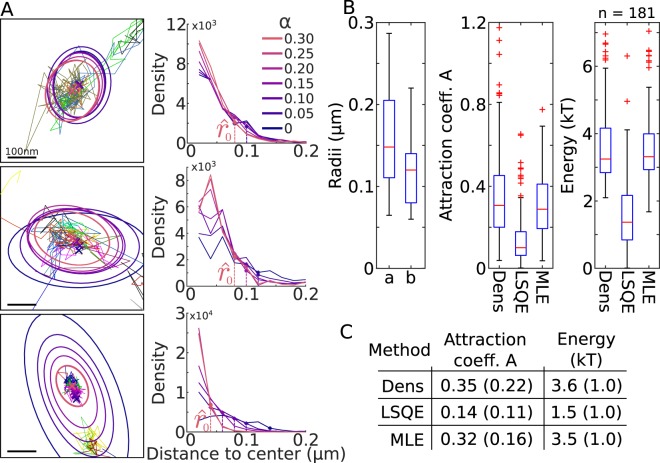
Reconstructed wells associated to GPI-anchored GFP. (**A**) 3 examples of potential wells (left) obtained from the density analysis on SPTs. The boundary of the well are estimated from various level of density *α* (right). The estimated radius ${\hat{r}}_{0}$ is computed using a threshold *T* = 4% on the density. (**B**) Box plots of the two semi-axes *a* and *b* (of the ellipse), estimated over 181 detected wells, the coefficient *A* and the energy (in kT), compared for the Density, LSQ and MLE methods. (**C**) Table of mean and SD for the coefficient *A* and the energy.

We also apply our statistical methods to the case of GFP linked to the outer leaflet of the membrane by a GPI-anchor (Fig. 8A–C), which are considered to be non-interacting molecules. However, we found many hiqh-density regions (*N* = 181), which are characterized as potential wells. The elliptic axes are *a* = 158 ± 57 nm and *b* = 118 ± 39 nm, associated with an energy of *E* = 3.6 ± 1.0, 1.5 ± 1.0 and 3.5 ± 1.0 kT for the density, LSQ and MLE methods respectively. To conclude, although it is surprising to detect high-density regions in GPI-GFP SPTs, we found here that they can be characterized as potential wells. Possibly they correspond to places where local signaling complexes or other transmembrane proteins are present. The exact nature of these regions remain unclear and should be further investigated. rather

## Summary and Discussion

### Two statistical methods to interpret high-density regions

We presented here two methods to extract the biophysical characteristics of high-density regions explored by SPTs. Interestingly, these regions are associated with bounded potential wells. The first method exploits the density of points of the trajectories, ignoring the causality between the successive points. It assumes that the nanodomain is a parabolic potential well with an elliptic base and a constant diffusion coefficient. In that case, the distribution of points inside the well is given by a Boltzmann distribution and should be uniform outside. We use this key observation to recover the main physical parameters and the location of the boundary. We compared also our result to the classical MLE (see Figs. S1, S2). The second method is based on estimating the vector field distribution at a given bin resolution Δ*x*. We used an optimal estimator to recover the characteristic of the field and we found that the boundary is located at the discontinuity between the converging field of the well (Ornstein-Ulhenbeck) and the random field generated the surrounding Brownian motion. Finally, the present methods are based on multiple averaging over many trajectories^21^, which provide robustness, reducing the effect of tracking errors or localization noise^24,25^.

The two methods are complementary and provide certain advantages compared to the MLE and PCA. In all cases, the center of the well could be retrieved. The quality of the estimators of the covariance parameters, however, were dependent on the method: changing the time Δ*t* and spatial Δ*x* steps influenced the recovery process as shown in Figs. 3, 4 and 6. The advantage of the first method is that we do not need to introduce an artificial grid of size Δ*x* which is a serious constraint in the second method as the bins size defines the resolution to recover the well and its boundary.

### High-density regions contained calcium-voltage channels and GPI SPTs data

We recall that high-density regions revealed by SPTs are not necessarily to due physical forces and potential wells^3^. However, for potential well, the geometry (center, curvature and boundary) can be recovered from our two methods. We applied them to CaV channels that mediate vesicular release at neuronal synapses and to phospholipid anchored GFP (GPI-GFP) moving on the cellular membrane. We found that the high-density regions for CaV (Fig. 7A) are characterized by two main axes with a length *a* = 104 ± 36 and *b* = 77 ± 20 nm (Fig. 7B), with a mean energy of 3.3 ± 0.8 kT (density method, Fig. 7C). We note that hydrogen bonds between calcium channels and phospholipid molecules could participate in the formation of the wells^26^. Surprisingly, we did not expect to find high-density regions for GPI-GFP, but we found several (Fig. 8A) that were characterized by average semi-axes lengths *a* = 158 ± 57 and *b* = 118 ± 39 nm (Fig. 8B), with a mean energy *E* = 3.6 ± 1.0 kT (density method, Fig. 8C). Possibly the higher energies of GPI-GFP wells can be due to the large variance caused by the lower number of trajectories restricted inside the wells as compared to CaV.

Although the interpretation of high-density regions as potential wells for AMPA receptors was first anticipated in^27^ and discovered in^3^, the nature of these wells and others, remains unclear^13^. Potential wells were found for membrane proteins such CaV^11^, GAG^12,28^ and recently for G-protein^29^. They could be generated by protein clusters, membrane cusps at vesicle fusion points or membrane-membrane contact at location of organelle interactions^30^. In general, potential wells are characterized by long-range forces of the order of hundreds of nanometers.

The wells could have multiple roles: they could retain receptors for hundreds of milliseconds to seconds at specific locations in order to increase the probability of a robust signal transduction, such as during synaptic transmission. Transient wells allow to trap proteins to create aggregates as proposed for capsid assembly^12,28^: once the energy of the well decreases, molecules are not interacting with the well anymore. Other possible roles for wells could be regulating the flow of receptors in micro-compartments such as dendritic spines^16^ or trapping proteins in the endoplasmic reticulum^11^. Finally, correlating undefined membrane geometry with an energy landscape remains difficult, because a physical model is needed to interpret them. Thus, the dynamics of receptors outside potential wells that deviates from trapped Brownian motion is still challenging to comprehend.

## Supplementary information

Supplementary Information
