# Supplementary material for: Biophysics of high density nanometer regions extracted from super-resolution single particle trajectories: application to voltage-gated calcium channels and phospholipids
Source: Sci Rep. 2019 Dec 11;9:18818. doi: 10.1038/s41598-019-55124-8 (PMC6906531; doi:10.1038/s41598-019-55124-8)
Supplement: Supplementary file 1 — Supplementary Information [file 41598_2019_55124_MOESM1_ESM.pdf]

Supplementary Information
Biophysics of high density nanometer regions extracted from super-resolution single particle trajectories: application to voltage-gated calcium channels and phospholipids

P. Parutto, J. Heck, M. Heine and D. Holcman *

October 18, 2019

In the SI, we first present the MLE estimator that we adapted to study potential wells. In the second part, we summarize some properties of the drift estimator that depends on the time step Δt and the bin size Δx.

1 MLE estimator for a potential well

We modified the Maximum likelihood Estimator (MLE) procedure to reconstruct from SPTs, the geometrical parameters (center and covariance matrix) of a well. Using a Ornstein-Ulhenbeck process, we apply the MLE procedure to the points of the trajectories falling inside the ensemble

$$\Gamma_\alpha = \{\boldsymbol{X}_i \,\text{such that}\, \rho(\boldsymbol{x}) > \alpha\}, \tag{1}$$

where ρ is the steady-state probability density function of the OU process. The advantage of the Maximum-likelihood approach is that no spatial discretization is needed. We recall that the transition probability density of an OU process centered at μ, with diffusion coefficient D and spring constant λ

$$\dot{x} = -\lambda(x-\mu) + \sqrt{2D}\dot{\omega} \tag{2}$$

*Ecole Normale Supérieure, 46 rue d'Ulm 75005 Paris, France and DAMPT, University Of Cambridge, Churchill College CB30DS, United Kingdom.

is

$$p(x,t|y,s) = \sqrt{\frac{\lambda}{2\pi D(1-e^{-2\lambda(t-s)})}} e^{-\frac{\lambda}{2D}\frac{(x-\mu-(y-\mu)e^{-\lambda(t-s)})^2}{(1-e^{-2\lambda(t-s)})}}. \quad (3)$$

The transition probability is

$$p(y_{i+1}|y_i) = \sqrt{\frac{\lambda}{2\pi D(1-e^{-2\lambda\Delta t})}} e^{-\frac{\lambda}{2D}\frac{(y_{i+1}-y_i e^{-\lambda\Delta t})^2}{1-e^{-2\lambda\Delta t}}}, \quad (4)$$

where a trajectory is discretized in $y_1, \dots, y_M$, with a fixed time step Δt. The log likelihood is

$$\begin{aligned} l(y_1,\dots,y_M|\lambda,\mu,D) &= \sum_{i=0}^{M-1} \log p(y_{i+1}|y_i) & (5)\\ &= \frac{M}{2}\log\frac{\lambda}{2\pi D(1-e^{-2\lambda\Delta t})} - \frac{\lambda}{2D(1-e^{-2\lambda\Delta t})}\sum_{i=0}^{M-1}(y_{i+1}-y_i e^{-\lambda\Delta t})^2. & (6)\end{aligned}$$

The maximum-likelihood approach consists in estimating λ that maximizes the log-likelihood $l(y_1, \dots, y_M)$. We change variables $x = e^{-\lambda\Delta t}$ and $v = \frac{\lambda}{2\pi D(1-e^{-2\lambda\Delta t})}$ so that

$$\tilde{l}(y_1,\dots,y_M|x,\mu,v) = \frac{M}{2}\log\frac{v}{\pi} - v\sum_{i=1}^{M-1}(y_{i+1}-\mu-(y_i-\mu)x)^2. \quad (7)$$

At the maximum,

$$\frac{\partial\tilde{l}}{\partial x} = \frac{\partial\tilde{l}}{\partial v} = \frac{\partial\tilde{l}}{\partial \mu} = 0, \quad (8)$$

leads to the coupled equations

$$\begin{aligned} \hat{\mu} &= \frac{\sum_0^{M-1} y_{i+1} - \hat{x}y_i}{M(1-\hat{x})} \\ \hat{x} &= \frac{\sum_0^{M-1}(y_{i+1}-\hat{\mu})(y_i-\hat{\mu})}{\sum_0^{M-1}(y_i-\hat{\mu})^2} \quad (9)\\ \hat{v} &= \frac{M}{2}\frac{1}{\sum_0^{M-1}((y_{i+1}-\hat{\mu})-(y_i-\hat{\mu})\hat{x})^2}. \end{aligned}$$

This system of equation can be solved leading to the following estimators [2]

$$\begin{aligned}
\hat{x} &= \frac{\frac{1}{M}\sum_{i=0}^{M-1} y_{i+1}y_i - \frac{1}{M^2}\sum_{i=0}^{M-1} y_{i+1}\sum_{i=0}^{M-1} y_i}{\frac{1}{M}\sum_{i=1}^{M-1} y_i^2 - \frac{1}{M^2}\left(\sum_{i=1}^{M-1} y_i\right)^2} + \frac{4}{M} \\
\hat{\mu} &= \frac{\frac{1}{M}\sum_{i=1}^{M-1}(y_{i+1} - \hat{x}y_i)}{1-\hat{x}}. \qquad (10)
\end{aligned}$$

The diffusion coefficient can be found from the third equation of eq. (9):

$$\hat{v} = \frac{1}{M}\sum_{0}^{M-1}\left(y_{i+1} - \hat{x}y_i - \hat{\mu}(1-\hat{x})\right)^2. \qquad (11)$$

This procedure can be generalized in two dimensions and in addition, we apply the estimator of eq. (9) to the ensemble Γ_α, defined by (1). We thus obtain the following estimators

$$\begin{aligned}
\hat{x}^\alpha &= \frac{\frac{1}{M_\alpha}\sum_{y_i\in\Gamma_\alpha} y_{i+1}y_i - \frac{1}{M_\alpha^2}\sum_{y_i\in\Gamma_\alpha} y_{i+1}\sum_{y_i\in\Gamma_\alpha} y_i}{\frac{1}{M_\alpha}\sum_{y_i\in\Gamma_\alpha} y_i^2 - \frac{1}{M^2}\left(\sum_{y_i\in\Gamma_\alpha} y_i\right)^2} + \frac{4}{M_\alpha} \\
\hat{\mu}^\alpha &= \frac{\frac{1}{M_\alpha}\sum_{y_i\in\Gamma_\alpha}(y_{i+1} - \hat{x}y_i)}{1-\hat{x}}, \qquad (12)
\end{aligned}$$

where M_α is the number of points $y_i \in \Gamma_\alpha$. We apply this estimator to numerical simulations in Figs. S1 and S2 and to CaV2.2 and GPI-GFP SPTs in Figs. 7 and 8 (main text) respectively.

2 Least Square Quadratic Estimator (LSQE)

2.1 Estimating the center and the curvature of the well

To recover the potential well from the drift distribution, we use a least square estimator

$$
\begin{aligned}
Err_{\boldsymbol{b}}(\mu_x, \mu_y, \lambda_x, \lambda_y) &= \sum_{i=1}^{N} \| - \nabla U(X_i) - \boldsymbol{b}(X_i)\|^2 \qquad (13) \\
&= \sum_{i=1}^{N} \left(\boldsymbol{b}_x^i + \lambda_x(x_i - \mu_x)\right)^2 + \left(\boldsymbol{b}_y^i + \lambda_y(y_i - \mu_y)\right)^2,
\end{aligned}
$$

where N is the number of points $\boldsymbol{X}_i = (x_i, y_i)$ and the potential well is

$$U(\boldsymbol{X}) = \lambda_x(x - \mu_x)^2 + \lambda_y(y - \mu_y)^2, \qquad (14)$$

so that

$$\boldsymbol{b}(\boldsymbol{X}) = -\nabla U(\boldsymbol{X}) = 2 \begin{bmatrix} \lambda_x(x - \mu_x) \\ \lambda_y(y - \mu_y) \end{bmatrix}. \qquad (15)$$

The minimizers are given by

$$\frac{\partial}{\partial \lambda_x} Err_{\boldsymbol{b}} = \frac{\partial}{\partial \lambda_y} Err_{\boldsymbol{b}} = 0 \qquad (16)$$

and

$$\frac{\partial}{\partial \mu_x} Err_{\boldsymbol{b}} = \frac{\partial}{\partial \mu_y} Err_{\boldsymbol{b}} = 0 \qquad (17)$$

from which, we obtain the center

$$\tilde{\mu}_x = \frac{\sum_{i=1}^{N} \boldsymbol{b}_x^i + \lambda_x x_i}{N\lambda_x}, \qquad \tilde{\mu}_y = \frac{\sum_{i=1}^{N} \boldsymbol{b}_y^i + \lambda_y y_i}{N\lambda_y}. \qquad (18)$$

and the eigenvalues of the covariance matrix:

$$\tilde{\lambda}_x = \frac{\sum_{i=1}^{N} \boldsymbol{b}_x^i (x_i - \mu_x)}{\sum_{i=1}^{N} (x_i - \mu_x)^2}, \qquad \tilde{\lambda}_y = \frac{\sum_{i=1}^{N} \boldsymbol{b}_y^i (y_i - \mu_y)}{\sum_{i=1}^{N} (y_i - \mu_y)^2}. \qquad (19)$$

In practice, we computed the center μ_x, μ_y and the eigenvalues λ_x, λ_y over the points $\boldsymbol{X}_i$ falling inside the well.

2.2 Center location and semi-axes from optimal fit

We derive here a close formula for the eigenvalues and the center associated to the optimal estimators of the drift. For an OU process, we recall that the eigenvalues are given by

$$\begin{aligned}\lambda_y &= \frac{\sum_{i=1}^{N} \boldsymbol{b}_y^i (y_i - \mu_y)}{\sum_{i=1}^{N} (y_i - \mu_y)^2} = \frac{\sum_{i=1}^{N} \boldsymbol{b}_y^i y_i - \mu_y \sum_{i=1}^{N} \boldsymbol{b}_y^i}{\sum_{i=1}^{N} y_i^2 - 2\mu_y \sum_{i=1}^{N} y_i + N\mu_y^2} \\ &= \frac{A_y - B_y \mu_y}{C_y - 2\mu_y D_y + N\mu_y^2}, \end{aligned} \tag{20}$$

where $A_y = \sum_{i=1}^{N} \boldsymbol{b}_y^i y_i$, $B_y = \sum_{i=1}^{N} \boldsymbol{b}_y^i$, $C_y = \sum_{i=1}^{N} y_i^2$ and $D_y = \sum_{i=1}^{N} y_i$. Using

$$\mu_y = \frac{\sum_{i=1}^{N} \boldsymbol{b}_y^i + \lambda_y \sum_{i=1}^{N} y_i}{N\lambda_y} = \frac{B_y + \lambda_y D_y}{N\lambda_y}, \tag{21}$$

and eq. (20) in eq. (21), we obtained

$$\mu_y = \frac{B_y + \frac{A_y - B_y \mu_y}{C_y - 2\mu_y D_y + N\mu_y^2} D_y}{N \frac{A_y - B_y \mu_y}{C_y - 2\mu_y D_y + N\mu_y^2}} \tag{22}$$

where μ_y is solution of the quadratic equation

$$2NB_y\mu_y^2 - (NA_y + 3B_yD_y)\mu_y + B_yC_y + A_yD_y = 0. \tag{23}$$

With $\Delta_y = N^2A_y^2 - 2NA_yB_yD_y + 9B_y^2D_y^2 - 8NB_y^2C_y$, we retain the positive solution

$$\mu_y = \frac{(NA_y + 3B_yD_y) + \sqrt{\Delta_y}}{4NB_y}. \tag{24}$$

Similarly, we get

$$\mu_x = \frac{(NA_x + 3B_xD_x) + \sqrt{\Delta_x}}{4NB_x} \tag{25}$$

Relations (24) and (25) lead to a close expression of the eigenvalues (eq. (19)).

2.3 Comparing MLE with density estimators

To recover the center and eigenvalues of an Ornstein-Uhlenbeck process with the potential well given in eq. (14), we apply the MLE procedure (section 1) for various values of the parameter α. We compare the results with the density (section 3 main text) and the LSQ (section 2) methods. We find that the MLE and density approaches are quite robust and give similar results, as shown in Fig. S1: Interestingly, all three estimators allow to recover the center (μ_x, μ_y) with high accuracy for the disk and the ellipse, when 100% to 50% of the points are taken into account $\alpha \in [0; 0.5]$ (Fig. S1). However, the estimation of the eigenvalues is acceptable for the MLE only, in the range $\alpha \in [0; 0.5]$, because it diverges in the two other cases, except when α 0.1 (90% of the distribution is used).

When the time step δt of the numerical simulations of eq. (2) and the sampling time Δt are equal, the three methods lead to a good recovery of the center $\hat{\mu}_x, \hat{\mu}_y$ (Fig. S2), but differ for recovering the eigenvalues $(\hat{\lambda}_x, \hat{\lambda}_y)$. The least square approach is less dependent on the parameter α than the two others. Probably because the distribution was generated with a large time step so that the statistics are calculated on trajectories far from the equilibrium. This result shows that the least square approach does not require to sample over a steady state distribution and thus recovering the parameters from the drift is possible for a large range of the parameter α. In section 4, we will estimate the effect of changing the time steps.

3 Influence of the time and spatial discretizations on the Least Square Estimation

In this section, we shall estimate the impact of the initial points distribution of the trajectories on the estimation of the drift. For the stochastic equation [7]

$$\dot{X} = \boldsymbol{A}(\boldsymbol{X}) + \sqrt{2}B(\boldsymbol{X})\dot{W}, \tag{26}$$

the optimal estimator for the drift $\boldsymbol{A}$ at a time resolution Δt is obtained by the formula [8, 5],

$$\begin{aligned} a_{\Delta t}(x) &= \mathbb{E}\left[\frac{X_{n+1} - X_n}{\Delta t} | X_n = x\right] = \frac{1}{\Delta t}\int_{\mathbb{R}} (y - x) p(X_{n+1} = y | X_n = x) dy \\ &= a(x) + o(1), \end{aligned} \tag{27}$$

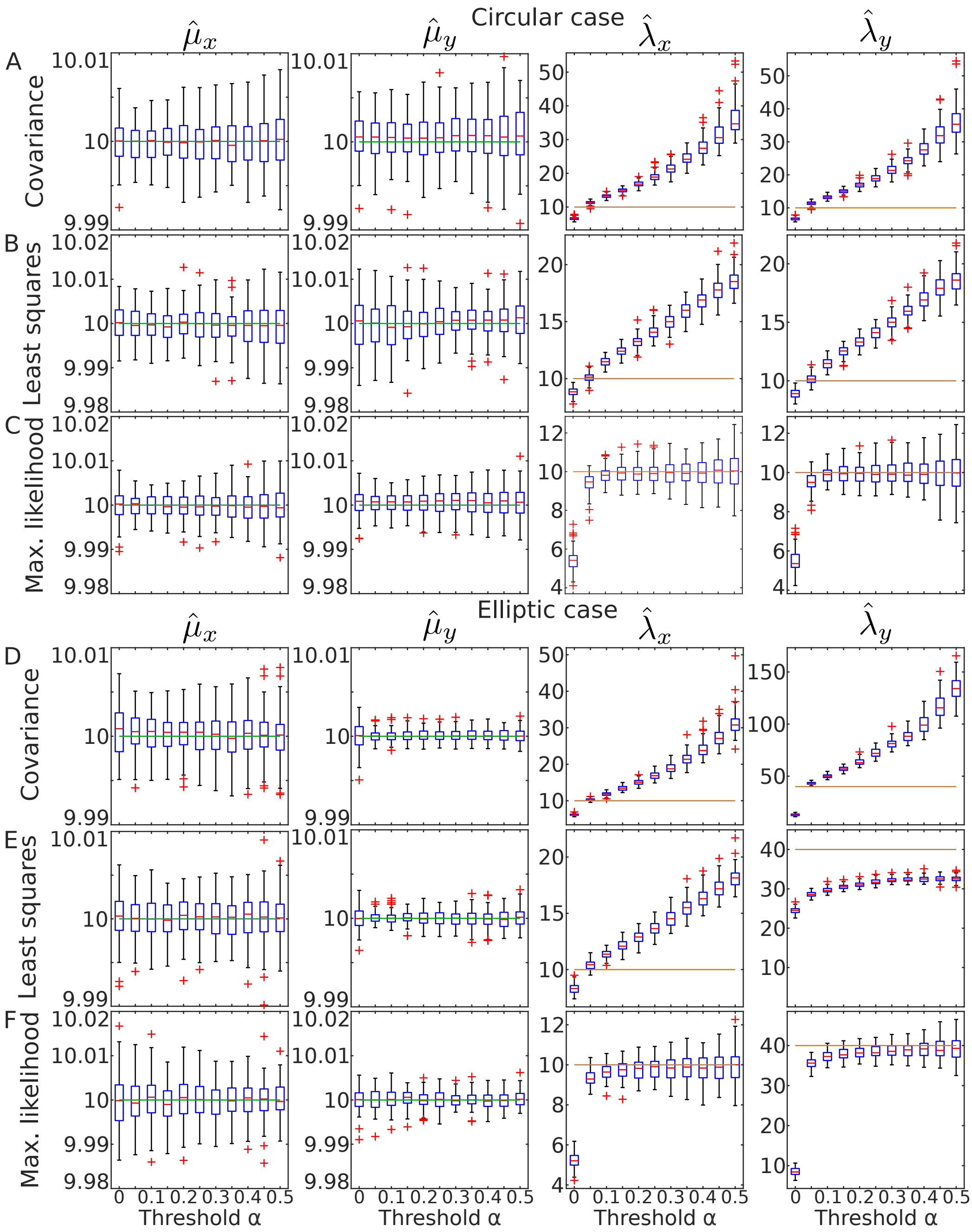

Figure S1: Comparing MLE vs covariance estimator. **A-B** estimation of λ_x, λ_y for the case of a disk for the MLE and covariance estimator respectively. The true value of $\lambda_x = \lambda_y = 10$ corresponds to the purple line. **C-D** same as in A-B for the case of an ellipse with $\lambda_x = 10$ and $\lambda_y = 40$. Data were obtained from stochastic simulations with a time step $\delta t = 10^{-4}$s whereas the sampling time was $\Delta t = 0.02s$.

have

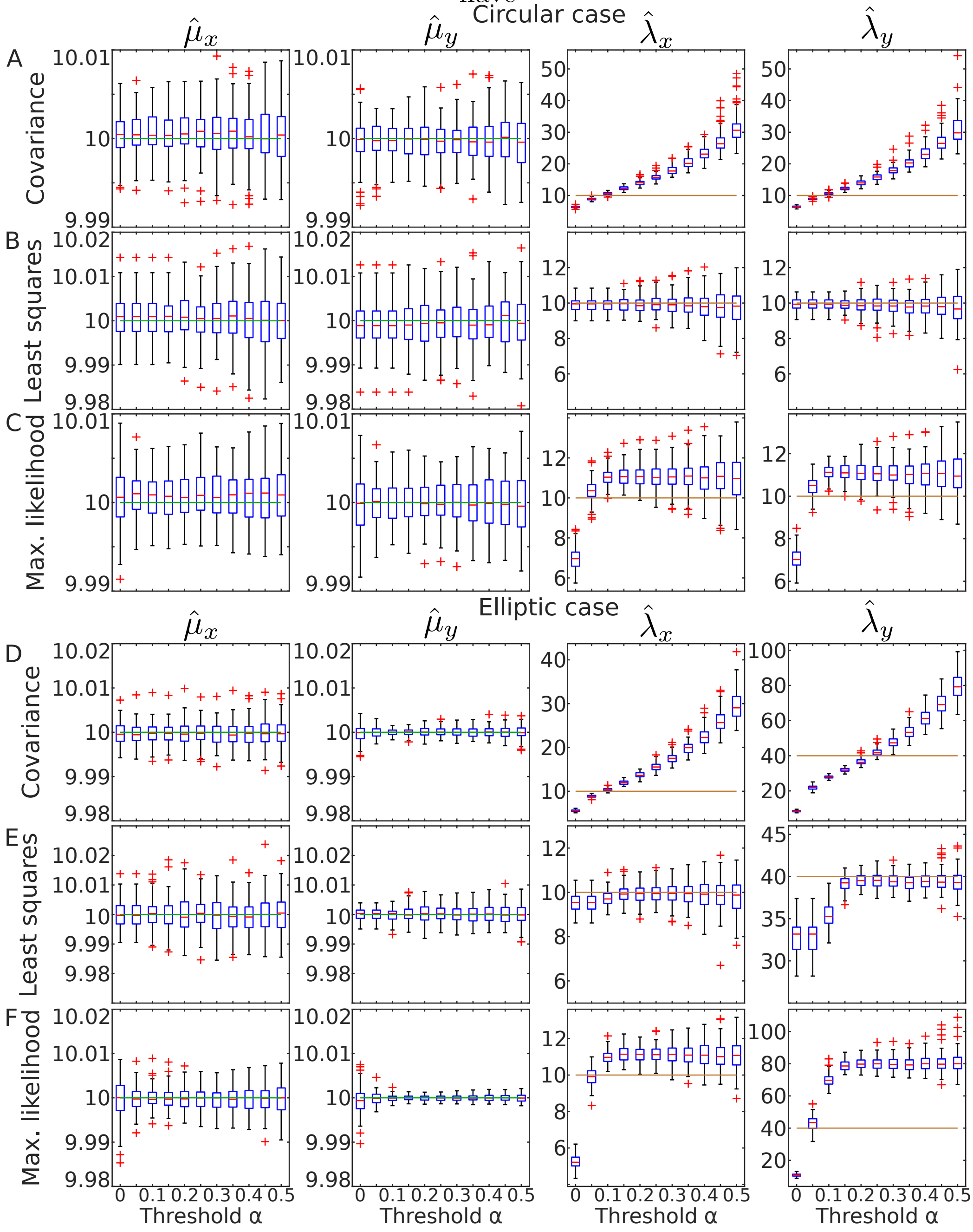

Figure S2: **Comparing MLE vs covariance estimator.** Same as Fig. S1 except that the simulation and sampling time steps are equal $\delta t = \Delta t = 0.02s$: **A-B** estimation of λ_x, λ_y for the case of a disk for the MLE and covariance estimator respectively. The true value of $\lambda_x = \lambda_y = 10$ corresponds to the purple line. **C-D** same as in A-B for the case of an ellipse with $\lambda_x = 10$ and $\lambda_y = 40$.

where $X_n = X(n\Delta t)$ and $\mathbb{E}[.]$ in eq. (27) is the expectation. When a grid of size Δx is used to estimate the drift map, all points in bin k leads to the same drift $a_{\Delta t}(x_k)$, where x_k is the center of the bin. For long trajectories, the stochastic process samples the steady-state distribution $p(\boldsymbol{x})$. Such distribution might influence the computation of the drift inside bin k. To estimate this contribution, we normalize the steady-state distribution $q(\boldsymbol{x})$ of the stationary process by

$$\begin{aligned} q_{\Delta x}(\boldsymbol{x}) &= Pr\{X \in x + \Delta x | X \text{ has a steady state } p(\boldsymbol{x})\} \\ &= \frac{p(\boldsymbol{x})}{\int_{\Delta(x)} p(\boldsymbol{x}) d\boldsymbol{x}}, \end{aligned} \tag{28}$$

where $\Delta(x) = [x - \Delta x/2, x + \Delta x/2]$. Note that $q_{\Delta x}(\boldsymbol{x}) \to \delta(\boldsymbol{x})$, when $\Delta x \to 0$. The estimated drift $a_{\Delta t}(x)$ depends on the distribution of points falling into the bin $\Delta(x)$ as follows

$$a_{\Delta t}(x)\Delta t + o(\Delta t) = \lim_{N\to\infty} \sum_{k=1}^{N} \mathbb{E}\left[Y_{n+1}^k - Y_n^k | Y_n^k \in \Delta(x)\right] \tag{29}$$

$$= \int_{\Delta_k} \int_{\mathbb{R}} (y - x) p_{\Delta t}(y|x) q_{\Delta x}(x) dy dx, \tag{30}$$

where $p_{\Delta t}(y|x)$ is the pdf to find $\boldsymbol{X}(t)$ at the point y at time $t + \Delta t$ when it started at point x at time t. In the small time limit,

$$\begin{aligned} a_{\Delta x}(x) = \lim_{\Delta t \to 0} \mathbb{E}\left[\frac{Y_{n+1} - Y_n}{\Delta t} | Y_n \in \Delta_k\right] &= \int_{\Delta_k} a(\boldsymbol{x}) q(\boldsymbol{x}) d\boldsymbol{x} = \int_{\Delta_k} a(x) \frac{p(x)}{\int_{x-\Delta x/2}^{x+\Delta x/2} p(x) dx} \\ &= \frac{\int_{\Delta(x)} p(y) a(y) dy}{\int_{\Delta(x)} p(y) dy}. \end{aligned}$$

We shall now obtain a further approximation by using a Taylor's expansion of the function $F(x) = \int_0^x p(s) a(s) ds$. We obtain that

$$\int_{\Delta_k} p(x) a(x) dx = F(x - \Delta x/2) - F(x + \Delta x/2) = F'(x)(\Delta x) + \frac{2}{6} F^{(3)}(x)(\Delta x/2)^3 + o((\Delta x)),$$

which leads to the approximation:

$$a_{\Delta x}(x) = \frac{p(x) a(x) \Delta x + \frac{1}{3}(p(x) a(x))''(x)(\Delta x/2)^3}{p(x)\Delta x + \frac{1}{3} p''(x)(\Delta x/2)^3} \tag{31}$$

$$= a(x)\left(1 + \left(\frac{a''(x)}{a(x)} + 2\frac{p'(x) a'(x)}{a(x) p(x)}\right)\frac{(\Delta x)^2}{24} + o((\Delta x)^2)\right). \tag{32}$$

The formula in higher dimension is given by

$$a_{\Delta x}(x) = a(x)\left(1 + \left(\frac{\Delta a(x)}{a(x)} + 2\frac{\nabla p(x).\nabla a(x)}{a(x)p(x)}\right)\frac{(\Delta x)^2}{24} + o((\Delta x)^2)\right). \quad (33)$$

We conclude that a discretization in bins of size Δx perturbs the drift recovery by a term $(\Delta x)^2$.

3.1 Influence of the time discretization Δt on the drift estimation

To study the consequences of a discrete sampling time on the reconstruction of the drift from SPTs, we focus on the one-dimensional OU-process

$$dX = -\lambda(X - \mu)dt + \sqrt{2D}dW. \quad (34)$$

where λ, μ are fixed. A direct integration of equation (34) for $s \leq t$ leads to

$$x(t) = x(s)e^{-\lambda(t-s)} + \mu(1 - e^{-\lambda(t-s)}) + e^{-\lambda(t-s)}\int_s^t \sqrt{2D}e^{\lambda u}dW_u. \quad (35)$$

and for two consecutive points $x(t)$ and $x(t + \Delta t)$, we have

$$x(t + \Delta t) - x(t) = -(x(t) - \mu)(1 - e^{-\lambda\Delta t}) + e^{-\lambda\Delta t}\int_t^{t+\Delta t} \sqrt{2D}e^{\lambda u}dW_u. \quad (36)$$

Thus, when Δt is small, the drift at position x and resolution Δt is

$$\begin{aligned} a_{\Delta t}(x)\Delta t + o(\Delta t) &= E\Big(x(t + \Delta t) - x(t)|x(t) = x\Big) \\ &= \int_{\mathbb{R}} (y - x)p(x(t + \Delta t) = y|x(t) = x)dy \\ &= -(1 - e^{-\lambda\Delta t})(x - \mu). \end{aligned} \quad (37)$$

We conclude that at resolution Δt, the approximation error is

$$F(t) = \frac{1 - e^{-\lambda\Delta t}}{\lambda\Delta t} = 1 - \frac{\lambda}{2}\Delta t + o(\Delta t) \quad (38)$$

suggesting that the drift of an OU process is always under-estimated using the displacement estimator.

3.2 Time and space discretization for Ornstein-Ulhenbeck process

We shall now evaluate the cumulative effect of a temporal Δt and spatial Δx discretization on the recovery of an OU-process. The spatial grid of size Δx and the drift at position x are estimated empirically using the points falling in the bin $\Delta(x) = [x - \Delta x/2, x + \Delta x/2]$. We start with the conditional steady-state distribution $q_\Delta(x)$ of points falling in $\Delta(x)$, which is linked to the pdf $p(x)$ of the OU-stationary process by

$$q_\Delta(x) = \frac{p(x)}{\int_{\Delta(x)} p(y)dy}. \tag{39}$$

The drift term from eq. (37) can be approximated as

$$\begin{aligned} a_{\Delta t,\Delta x}(x) &= \mathbb{E}\left(\frac{x(t+\Delta t) - x(t)}{\Delta t}|x(t) \in \Delta(x)\right) \\ &= \int_\Delta \int_{\mathbb{R}} \frac{y-x}{\Delta t} p(x(t+\Delta t) = y|x(t) = x)dydx \\ &= -\frac{1-e^{-\lambda \Delta t}}{\Delta t} \int_{\Delta(x)} (x-\mu) q_\Delta(x) dx, \end{aligned} \tag{40}$$

where μ is the center of the OU-process and the stationary pdf is given by

$$p(x) = \sqrt{\frac{\lambda}{2\pi D}} e^{-\dfrac{\lambda}{2D}(x-\mu)^2}. \tag{41}$$

To estimate eq. (40), we use eq. (31). For this computation, we set $\mu = 0$. In that case, we have

$$\int_{\Delta(x)} y q_{\Delta(x)}(y) dy = \frac{\int_{\Delta(x)} y e^{-\frac{\lambda}{2D} y^2} dx}{\int_{\Delta(x)} e^{-\frac{\lambda}{2D} y^2} dy}. \tag{42}$$

In the small Δx approximation, we have

$$\begin{aligned}
\int_\Delta y q_\Delta(y)dy &= x + \frac{\int_{-\Delta x/2}^{\Delta x/2} h e^{-\frac{\lambda}{2D}(x+h)^2} dh}{\int_{-\Delta x/2}^{\Delta x/2} e^{-\frac{\lambda}{2D}(x+h)^2} dh} \\
&= x + \frac{\int_{-\Delta x/2}^{\Delta x/2} h e^{-\frac{\lambda}{2D}h^2} e^{-\frac{\lambda}{D}xh} dh}{\int_{-\Delta x/2}^{\Delta x/2} e^{-\frac{\lambda}{2D}h^2} e^{-\frac{\lambda}{D}xh} dh} \\
&= x + \frac{-\frac{\lambda}{4D}x(\Delta x)^3 + o((\Delta x)^3)}{\Delta x + (\frac{\lambda^2 x^2}{D^2} - \frac{\lambda}{D})(\Delta x)^3 + o(\Delta x^3)} \\
&= x + \frac{-\frac{\lambda}{6D}x\Delta x^2 + o((\Delta x)^2)}{1 + (\frac{\lambda^2 x^2}{D^2} - \frac{\lambda}{D})(\Delta x)^2 + o((\Delta x)^2)} \\
&= x(1 - \frac{\lambda}{12D}(\Delta x)^2) + o((\Delta x)^2). \qquad (43)
\end{aligned}$$

Using eqs. (40) and (43), we obtain an approximation for the drift at finite time step Δt and grid size Δx

$$a_{\Delta t,\Delta x}(x) = -\frac{1 - e^{-\lambda \Delta t}}{\Delta t}(x - \mu - \frac{\lambda}{12D}(\Delta x)^2 + o((\Delta x)^2)). \qquad (44)$$

To conclude, relation (44) reveals that the empirical displacements $x(t + \Delta t) - x(t)$ collected over trajectories for an OU, can be used to recover the drift, with an additional exponential order correction in Δt and a second order in Δx.

3.3 Empirical estimations of the drift

The empirical estimator $\tilde{a}$ of the drift at position x for finite time Δt and spatial steps Δx, is defined

$$\tilde{a}_{\Delta t,\Delta x}(x) = \frac{1}{N}\sum_{i=1}^{N} \sum_{x^i(t_j)\in\Delta(x)} \frac{x^i(t_{j+1}) - x^i(t_j)}{\Delta t}, \qquad (45)$$

where N is the number of points $x^i(t_j)$ located in the square bin of infinitesimal surface $(\Delta x)^2$ around x. Using eq. (44), the approximation at second order in Δx gives that

$$\tilde{a}(x) = -\frac{1 - e^{-\lambda \Delta t}}{\Delta t}(x_k - \mu - \frac{\lambda}{12D}\Delta x^2) + o(\Delta x^2). \qquad (46)$$

To recover the parameter λ at various order of Δx, we can express $\tilde{\lambda}$ using a regular series expansion

$$\tilde{\lambda}(\Delta t) = \lambda_0 + \lambda_2(\Delta x)^2 + \lambda_4(\Delta x)^4 + ... \tag{47}$$

The first term is obtained by setting $\Delta x = 0$ in eq. (46), leading for any bin centered around x_k for $k = ..N_b$ to

$$\tilde{a}(x_k) = -\frac{1 - e^{-\lambda_0 \Delta t}}{\Delta t}(x_k - \mu). \tag{48}$$

In that case, a linear regression method can be used using the two coordinates $x_k - \mu$ and $\tilde{a}$ and to fit the distribution with a line and invert eq. (48). If necessary, the next in the expansion can be found. Note that a formal inversion of eq. (48) shows that for each k, we can obtain an estimation for λ_0:

$$\lambda_0^k(\Delta t) = -\frac{1}{\Delta t}\log(1 - \frac{\tilde{a}(x_k)\Delta t}{x_k - \mu}). \tag{49}$$

so that

$$\hat{\lambda}_0(\Delta t) = -\sum_{k=1}^{N_b} \frac{1}{\Delta t}\log(1 - \frac{\tilde{a}(x_k)\Delta t}{x_k - \mu}), \tag{50}$$

showing that numerical fluctuations in $\tilde{a}(x_k)$ for $|x_k - \mu|$ small can drastically affect the estimation. We use this result to study the recovery of the parameters in Fig. 5C (main text) and Fig. S3C, where we indeed observe larger errors near the center of the well than inside. We refer to Fig. 6 (Main text) for the estimation of the eigenvalue with and without the center bin.

3.4 Effect of the grids intersecting the boundary in the estimation of the drift

The recovery of a truncated OU involves estimating several parameters that depend on the accurate detection of the boundary. We focus here on the drift estimation for the part of the square grid that intersects the boundary (green bins in Fig. 6, main text). In that case, for the interior part that intersects the elliptic domain, the empirical estimation recovers the local vector, while outside, it fluctuates around zero, due to the nature of the Brownian motion

(no drift). Thus the error of the drift estimation at the boundary increases with the area fraction of the bin falling outside the domain. To estimate this error, we recall that the truncated OU-process is defined by

$$\dot{X} = -\nabla U(\boldsymbol{X})dt + \sqrt{2}B(\boldsymbol{X})\dot{W}, \tag{51}$$

where

$$U(\boldsymbol{X}) = \begin{cases} A\left[(\frac{x-\mu_x}{a})^2 + (\frac{y-\mu_x}{b})^2\right], & \text{if } \boldsymbol{X} \in \Gamma_{\mathcal{E}} \\ \mathcal{E} & \text{otherwise} \end{cases}. \tag{52}$$

Since the drift is zero for a diffusion process, located outside $\Gamma_{\mathcal{E}}$, we have

$$\begin{aligned} a_{\Delta x}(\boldsymbol{x}) &= \lim_{\Delta t \to 0} \mathbb{E}\left[\frac{Y_{n+1} - Y_n}{\Delta t} | Y_n \in \Delta(\boldsymbol{x}) \cap \Gamma_{\mathcal{E}}\right] \\ &= \int_{\Delta_k^1(\boldsymbol{x})} a(\boldsymbol{y})q(\boldsymbol{y})d\boldsymbol{y}, \end{aligned} \tag{53}$$

where $\Delta^1(\boldsymbol{x}) = \Delta(\boldsymbol{x}) \cap \Gamma_{\mathcal{E}}$ is the part of the grid interior to the ellipse. Indeed, the drift a diffusion process is zero. In addition, we suppose that the sample is made according to a normalized distribution

$$q_{\Delta x}(\boldsymbol{x}) = \frac{p(\boldsymbol{x})}{\int_{\Delta(x)} p(\boldsymbol{y})d\boldsymbol{y}}, \tag{54}$$

where $p(\boldsymbol{x})$ is any distribution that could be the steady-state distribution of a truncated OU inside the well and is uniform outside. From eq. (53), we get

$$a_{\Delta x}(\boldsymbol{x}) = a(\boldsymbol{x})\frac{\int_{\Delta^1(x)} p(\boldsymbol{x})d\boldsymbol{x}}{\int_{\Delta(x)} p(\boldsymbol{x})d\boldsymbol{x}}. \tag{55}$$

In first approximation,

$$a_{\Delta x}(\boldsymbol{x}) = a(\boldsymbol{x})\frac{\Delta^1(x)}{\Delta^1(x) + \Delta^2(x)}, \tag{56}$$

where $\Delta^2(x) = \Delta(\boldsymbol{x}) \cap \Gamma_{\mathcal{E}}\Delta(\boldsymbol{x})$. We shall now estimate $\Delta^1(x)$. We first note that the conservation of surfaces: $\Delta^1(x) + \Delta^2(x) = (\Delta x)^2$. For a square centered at a boundary of the ellipse (x, y)

$$\frac{x^2}{a^2} + \frac{y^2}{b^2} = 1, \tag{57}$$

we consider the square grid with integer coordinates $k = [\frac{x}{\Delta x}]$ and $q = [\frac{y}{\Delta x}]$. To compute $\Delta^2(x)$, we subtract the total area of the rectangle

$$S^{(2)} = [\Delta x][(q+1)\Delta x] \tag{58}$$

to the surface underneath the ellipse between the point $k\Delta x$ and $\Delta x + k\Delta x$:

$$S^{(1)} = \int_{k\Delta x}^{(k+1)\Delta x} b\sqrt{1-(\frac{u}{a})^2}du \tag{59}$$

$$\approx b\sqrt{1-\frac{k^2(\Delta x)^2}{a^2}}\left(1-\frac{1}{2}\frac{k\Delta x^2}{a^2}\right). \tag{60}$$

Thus the computations lead to

$$S^{(2)} - S^{(1)} = \frac{1}{2}\frac{k\Delta x^2}{a^2} + o(\Delta x^2) \tag{61}$$

and for $x > \Delta x$, we get

$$\Delta^2(x) = S^2 - S^1 = \tfrac{xy}{2a^2}(\Delta x)^2 + o(\Delta x^2). \tag{62}$$

A similar computation leads for $0 < x \leq \Delta x$ to

$$\Delta^2(x) = S^1 - S^2 = (\Delta x - x)y + o(\Delta x^2). \tag{63}$$

To conclude, except for the bin at the four extreme positions of the ellipse, the error is of order $O((\Delta x)^2)$ for each grid bin, leading to a cumulative error along the total length of $O((\Delta x))$. The error contribution is shown in Fig. 6E.

4 Influence of the time steps in stochastic simulations

In the Smoluchowski's limit of the Langevin equation, the first order stochastic equation from which trajectories are generated is obtained by choosing a time step δt. This time step should not be smaller than the reciprocal of the friction coefficient γ so that the successive points $X(\delta t), X(2\delta t), ..X(n\delta t), ...$ should approximate the physical trajectory. When the sampling rate is such that $\Delta t \gg \delta t$, we can compare the drift estimation in that case and also

study the extreme case when the sampling and simulation time steps are identical, leading to a jump process. When $\Delta t \gg \delta t$, the drift is computed after n steps. Using the empirical estimator, in the limit of a large number of trajectories N, we get

$$\begin{aligned} a^{est}_{\Delta t}(x)\Delta t + o(\Delta t) &= \lim_{N\to\infty} \frac{1}{N}\sum_{m=1}^{N}(x^m(t+p\delta t) - x^m(t+(p-1)\delta t)) + ...(x^m(t+\delta t) - x^m(x)) \\ &\approx \int_0^{n\delta t} \mathbb{E}[a(x(s))|x(0)=x]ds \\ &= \int_0^{\Delta t}\int_y a(y)p_s(y|x)dsdy, \end{aligned} \tag{64}$$

where $p_s(y|x)$ is the pdf of the process $X(t)$ starting at x at time 0 and ending at y at time s. This result is quite different from the classical estimator of eq. (29). We note that the result of eq. (64) is very different from the estimation from a single observation time step Δt. Using the pdf an Onrstein-Uhlenbeck process

$$p_s(y|x) = \sqrt{\frac{\lambda}{2\pi D(1-e^{-2\lambda s})}}\exp\{-\frac{\lambda}{2D}\frac{(y-xe^{-\lambda s})^2}{1-e^{-2\lambda s}}\}, \tag{65}$$

and the change of variable $u = \frac{(y-xe^{-\lambda s})^2}{\sqrt{\frac{2D}{\lambda}(1-e^{-2\lambda s})}}$ we get

$$\int_0^{\Delta t}\int_y a(y)p_s(y|x)dsdy = \int_0^{\Delta t}\int_u a(xe^{-\lambda s} + \sqrt{\frac{2D}{\lambda}(1-e^{-2\lambda s})}u)\frac{1}{\sqrt{\pi}}\exp\{-u^2\}duds. \tag{66}$$

Using a Taylor's expansion in the drift term:

$$\begin{aligned} a(xe^{-\lambda s} + \sqrt{\frac{2D}{\lambda}(1-e^{-2\lambda s})}u) &= \\ a(xe^{-\lambda s}) + \sqrt{\frac{2D}{\lambda}(1-e^{-2\lambda s})}ua'(xe^{-\lambda s}) &+ \frac{1}{2}\frac{2D}{\lambda}(1-e^{-2\lambda s})u^2a''(xe^{-\lambda s}) + o((1-e^{-2\lambda s}))^2. \end{aligned}$$

Thus the estimator of the drift with many time steps is at first order in Δt given by

$$\begin{aligned}\int_0^{\Delta t}\int_y a(y)p_s(y|x)dsdy &= \int_0^{\Delta t}\left(a(xe^{-\lambda s})+\frac{1}{2}\frac{D}{\lambda}(1-e^{-2\lambda s})a''(xe^{-\lambda s})\right)ds\\ &\approx a(x)\Delta t+(-\lambda x a'(x)+Da''(x))\frac{(\Delta t)^2}{2}+o((\Delta t)^2)\\ &\approx -\lambda x\Delta t+\lambda^2 x\frac{(\Delta t)^2}{2}+o((\Delta t)^2).\end{aligned}$$

We now evaluate the consequences of simulating a process with many time steps $\delta t = 10^{-4}$s whereas the sampling time was $\Delta t = 0.02s$ in Fig. 5 (main text). We show in Fig. S3 how the drift field can be recovered. This situation corresponds to large jumps of the underlying physical process. To conclude, we find that the center and peripheral grid bins are generating most of the error, especially for large grid sizes ($\Delta x = 50$ and 90 nm).

5 Conditional drift estimation

In this last section, we discuss the effect of estimating the drift by conditioning the end points of a displacement to stay inside the potential well. Computing the displacement ΔX by selecting only trajectories that stay inside the well gives a bias estimator of the drift. This situation appears when the trajectories never reach the boundary. The drift estimator is computed from the conditional pdf p^* of the process that stays inside the potential well. To find such a drift, we introduce the probability that a stochastic particle hits a ball of radius ϵ centered on the well before escaping from the well [9], then

$$p^*(x,y,t) = p(x,y,t)\frac{q(x)}{q(y)}, \tag{67}$$

where p is the pdf in the entire space. q is solution of

$$L^*(q) = 0 \tag{68}$$

$$q = 0 \text{ on } \partial W \tag{69}$$

$$q = 1 \text{ on } \partial B_\epsilon, \tag{70}$$

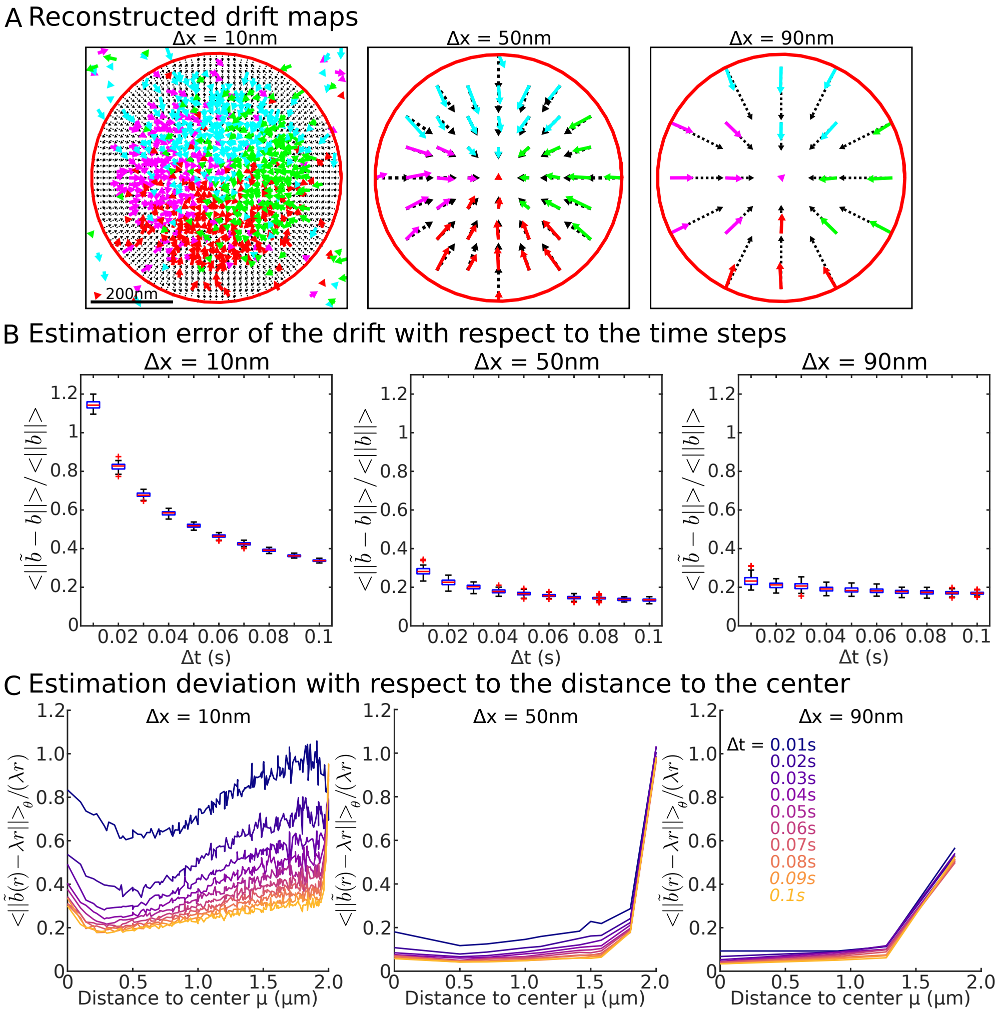

Figure S3: **Recovering the vector field with an equal simulated and sampled time step $\delta t = \Delta t$ A** Recovering the local drift field inside a circular well for different grid sizes (10 nm, 50 nm, 90 nm) using numerical simulations with a sampling $\Delta t = 20$ ms, with the constraints that at least 10 points falls inside a bin. **B** Error between the true and observed fields averaged over all the square bins inside the well versus the time step Δt. **C** Error between the true and observed fields averaged over the radial angle versus the distance r to the center for various timestep (see color code).

L^* is the backward Fokker-Planck equation associated the process $X(t)$ (p.77 [9]), defined by

$$d\boldsymbol{x} = \boldsymbol{A}(\boldsymbol{x})dt + \sqrt{2D}d\boldsymbol{w}. \tag{71}$$

In that case,

$$E\left(\frac{\boldsymbol{x}(t+\Delta t)-\boldsymbol{x}(t)}{\Delta t}|x(t)=x\right) = \boldsymbol{A}(x) + \sqrt{2D}\frac{\nabla q(\boldsymbol{x})}{q(\boldsymbol{x})}. \tag{72}$$

To conclude, by restricting the computation of the displacements to empirical trajectories that only remain in the well, an additional term has to be accounted for, which diverges as the distance from the point x to the boundary tends to zero (Fig. S4).

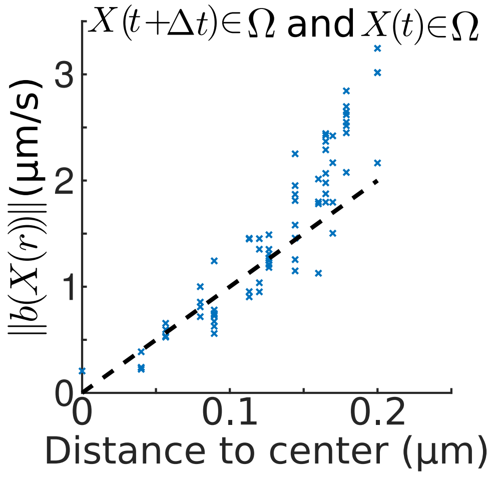

Figure S4: **Conditional reconstructed drift versus distance to the center.** We estimated the drift for displacements that do not exit the well (assuming the boundary is known).

References

[1] Hoze N, Holcman D, 2014. Residence times of receptors in dendritic spines analyzed by stochastic simulations in empirical domains. *Biophys. J.* 107:3008-17.

[2] Tang C.Y. and Chen S.X. (2009). "Parameter estimation and bias correction for diffusion processes", Journal of Econometrics, vol. 149, p. 65-81.

[3] Holcman, D., Schuss, Z. (2017). 100 years after Smoluchowski: stochastic processes in cell biology. Journal of Physics A: Mathematical and Theoretical, 50(9), 093002.

[4] Hoze, N., Holcman, D. (2017). Coagulation-Fragmentation with a Finite Number of Particles: Models, Stochastic Analysis, and Applications to Telomere Clustering and Viral Capsid Assembly. In Stochastic Processes, Multiscale Modeling, and Numerical Methods for Computational Cellular Biology (pp. 205-239). Springer, Cham.

[5] N. Hozé and D. Holcman. Statistical methods for large ensembles of super-resolution stochastic single particle trajectories in cell biology. *Annual Review of Statistics and Its Application*, 4:189–223, 2017.

[6] Holcman D, Hoze N, Schuss Z, 2015. Analysis and interpretation of superresolution single-particle trajectories. *Biophys. J.*, 109:1761-1771.

[7] Hoze N, Holcman D, 2014. Residence times of receptors in dendritic spines analyzed by stochastic simulations in empirical domains. *Biophys. J.* 107:3008-17.

[8] Hoze N, Holcman D, 2015. Recovering a stochastic process from super-resolution noisy ensembles of single-particle trajectories. *Phys. Rev. E* 92 (5), 052109.

[9] Karlin S, Taylor HM, *A Second Course on Stochastic Processes* (Academic Press, 1981).

[10] Qian H, Sheetz MP, Elson EL, 1991. Single particle tracking. Analysis of diffusion and flow in two-dimensional systems. *Biophys. J.*, 60:910.

[11] Schuss Z, 2010. *Theory and Applications of Stochastic Processes: An Analytical Approach.* Applied Mathematical Sciences vol.170, Springer NY.

[12] Vestergaard CL, Blainey P, Flyvbjerg H, 2014. Optimal estimation of diffusion coefficients from single-particle trajectories. *Phys. Rev. E*, 89:022726.

[13] Vestergaard et al., 2015. Estimation of motility parameters from trajectory data. *The european physical journal special topics.*

[14] Z. Schuss, *Diffusion and Stochastic Processes: an Analytical Approach* (Springer, New York, 2010).
